# Type I Interferon Drives Dendritic Cell Apoptosis via Multiple BH3-Only Proteins following Activation by PolyIC *In Vivo*

**DOI:** 10.1371/journal.pone.0020189

**Published:** 2011-06-02

**Authors:** Silvia A. Fuertes Marraco, Clare L. Scott, Philippe Bouillet, Annette Ives, Slavica Masina, David Vremec, Elisa S. Jansen, Lorraine A. O'Reilly, Pascal Schneider, Nicolas Fasel, Ken Shortman, Andreas Strasser, Hans Acha-Orbea

**Affiliations:** 1 Department of Biochemistry, University of Lausanne, Epalinges, Switzerland; 2 The Walter and Eliza Hall Institute of Medical Research (WEHI), Melbourne, Australia; 3 Department of Medical Biology, University of Melbourne, Melbourne, Australia; Agency for Science, Technology and Research (A*STAR), Singapore

## Abstract

**Background:**

DC are activated by pathogen-associated molecular patterns (PAMPs), and this is pivotal for the induction of adaptive immune responses. Thereafter, the clearance of activated DC is crucial to prevent immune pathology. While PAMPs are of major interest for vaccine science due to their adjuvant potential, it is unclear whether and how PAMPs may affect DC viability. We aimed to elucidate the possible apoptotic mechanisms that control activated DC lifespan in response to PAMPs, particularly *in vivo*.

**Methodology/Principal Findings:**

We report that polyinosinic:polycytidylic acid (PolyIC, synthetic analogue of dsRNA) induces dramatic apoptosis of mouse splenic conventional DC (cDC) *in vivo*, predominantly affecting the CD8α subset, as shown by flow cytometry-based analysis of splenic DC subsets. Importantly, while Bim deficiency conferred only minor protection, cDC depletion was prevented in mice lacking Bim plus one of three other BH3-only proteins, either Puma, Noxa or Bid. Furthermore, we show that Type I Interferon (IFN) is necessary and sufficient for DC death both *in vitro* and *in vivo*, and that TLR3 and MAVS co-operate in IFNß production *in vivo* to induce DC death in response to PolyIC.

**Conclusions/Significance:**

These results demonstrate for the first time *in vivo* that apoptosis restricts DC lifespan following activation by PolyIC, particularly affecting the CD8α cDC subset. Such DC apoptosis is mediated by the overlapping action of pro-apoptotic BH3-only proteins, including but not solely involving Bim, and is driven by Type I IFN. While Type I IFNs are important anti-viral factors, CD8α cDC are major cross-presenting cells and critical inducers of CTL. We discuss such paradoxical finding on DC death with PolyIC/Type I IFN. These results could contribute to understand immunosuppression associated with chronic infection, and to the optimization of DC-based therapies and the clinical use of PAMPs and Type I IFNs.

## Introduction

Dendritic cells (DC) are the most potent professional antigen-presenting cells and play a key role in the induction of tolerance and initiation of adaptive immune responses [1]. Such decisive role of DC has attracted major attention for the improvement of vaccine efficacy, including the targeting of antigens to DC and the use of adjuvants to optimally activate DC and consequently boost immunity [2].

The DC system is composed of phenotypically and functionally heterogeneous subsets [1], [3]. Migratory DC reside in non-lymphoid organs and are able to capture antigens at the sites of infection or injury and transport them to draining lymph nodes. Lymphoid tissue-resident, conventional DC (cDC) are non-migratory and serve as sentinels in secondary lymphoid organs and tissues for *in situ* antigen capture and presentation. In the mouse, several DC subtypes can be found in the spleen: two subsets of cDC (CD8α^+^ CD11b^−^ and CD8^−^ CD11b^+^, hereafter termed CD8α and CD11b subsets), inflammatory monocyte-derived DC, and plasmacytoid DC (pDC).

Pattern-Recognition Receptors (PRR), including the Toll like receptor (TLR) family, detect Pathogen-Associated Molecular Patterns (PAMPs) and are important for the stimulation of antigen-presenting activity, featuring up-regulation of co-stimulatory molecules and the secretion of a variety of chemokines and cytokines [4]. Importantly, following detection of PAMPs, DC switch from tolerance induction to T cell priming. Notably, the various DC subsets differ in their profile of PRR expression as well as in their cytokine responses and antigen-presenting capacities, with a consequent division of labour amongst DC subsets in pathogen recognition and immune response initiation [4], [5]. Amongst the different PAMPs under research and used in clinical studies, the synthetic dsRNA analog polyinosinic:polycytidylic acid (PolyIC) has proven superiority as a vaccine adjuvant for Th1 immunity, including in DC targeted vaccines [2], with the induction of Type I Interferon (IFN) by PolyIC being key to the enhancement of DC function [6].

The control of DC lifespan is yet another critical parameter that can profoundly influence the outcome of an immune response, potentially affecting vaccine efficacy and its side-effects [7], [8]. While an increase in the lifespan of DC can elicit autoimmunity [9], reduced DC numbers correlate with immunosuppressed states in several settings both in humans and in mice, including chronic viral infection, sepsis and cancer [7]. The DC compartment must be strictly regulated, in order to sustain homeostasis, to return to steady-state levels following the induction of an immune response, to prevent autoimmunity, and to avoid immunosuppresion.

Apoptotic cell death is thought to play a critical role in the regulation of the numbers and function of DC [7], [8]. Mammals have two distinct but ultimately converging apoptosis signaling pathways. The “death receptor” (or “extrinsic”) pathway is triggered by members of the TNF-R family, containing intra-cellular “death domains” (e.g. Fas, TNF-R1), and requires activation of the initiator caspase, caspase-8. The “Bcl-2-regulated” (also called “intrinsic” or “mitochondrial”) apoptotic pathway can be triggered by developmental cues, cytokine withdrawal or cytotoxic stress signals (e.g. DNA damage); it involves mitochondrial outer membrane permeabilization (MOMP) and consequent release of apoptogenic factors, such as cytochrome c, which promotes activation of the initiator caspase-9, unleashing the caspase cascade. This pathway is controlled by the Bcl-2 family and these proteins can be sub-divided according to structure and function [10]. The anti-apoptotic members, including Bcl-2, Bcl-x_L_, Mcl-1 and A1, share up to 4 regions of homology (BH or Bcl-2 Homology domains) and are essential for cell survival. Bax and Bak have three BH regions and are critical for MOMP and activation of the effector phase of apoptosis. The BH3-only subgroup, including Bim, Bid, Puma and Noxa, is essential for initiation of apoptosis and thought to activate Bax/Bak proteins either directly or indirectly by sequestering their pro-survival relatives [11]. Both the “death receptor” and the “Bcl-2-regulated” apoptotic pathways converge upon the activation of the effector caspases, caspase-3, -6 and -7, which cleave a multitude of substrates and thereby cause cellular demolition. In addition, cross-talk between “intrinsic” and “extrinsic” apoptosis pathways may occur. For instance, caspase-8 may proteolytically activate the pro-apoptotic BH3-only member Bid that will then activate the “intrinsic”, mitochondrial death pathway.

The apoptotic mechanisms that regulate DC function are poorly understood. T-cell dependent killing of antigen-presenting DC has been described for both CD4 and CD8 T cells, via FasL and perforin secretion, respectively, as a negative feedback mechanism to attenuate immune responses [12], [13]. However, there is incomplete or contradictory evidence on the consequences of microbial stimulation for DC viability. Treatment of DC *in vitro* with TLR-Ls results in up-regulation of the pro-apoptotic protein Bim, but TLR-Ls rather promote survival and appear to have an inhibitory effect on the spontaneous apoptosis of DC in culture [14]. Furthermore, Bim-deficient DC are partially protected from spontaneous apoptosis *in vitro*, regardless of the presence or absence of TLR-Ls [14]. In contrast, treatments *in vivo* using TLR-Ls, such as CpG or LPS, as well as infection with E.coli or LCMV-Armstrong, induce loss of splenic cDC [15], [16]. To date, there is a lack of information on the possible apoptotic pathways that control DC populations during the early phases of activation *in vivo*, particularly in response to PAMPs and independently of other immune cells.

Here we show that PolyIC induces apoptosis of splenic cDC following their activation *in vivo*, with the CD8α cDC being more susceptible than the CD11b cDC subset. Proving the apoptotic mechanism involved *in vivo*, absence of Bim together with a second BH3-only family member (Bid, Noxa or Puma) leads to protection from DC death. We also show that Type I IFN production is required and sufficient for PolyIC-induced DC death. This study sheds light into the mechanisms of activated DC death *in vivo*. The results could contribute to an understanding of immunosuppression during chronic viral infection and inflammation, and may provide important clues for the optimization of vaccines and treatments featuring PAMPs and Type I IFNs.

## Results

### Splenic cDC are reduced in numbers following PolyIC treatment

**Figure 1:**
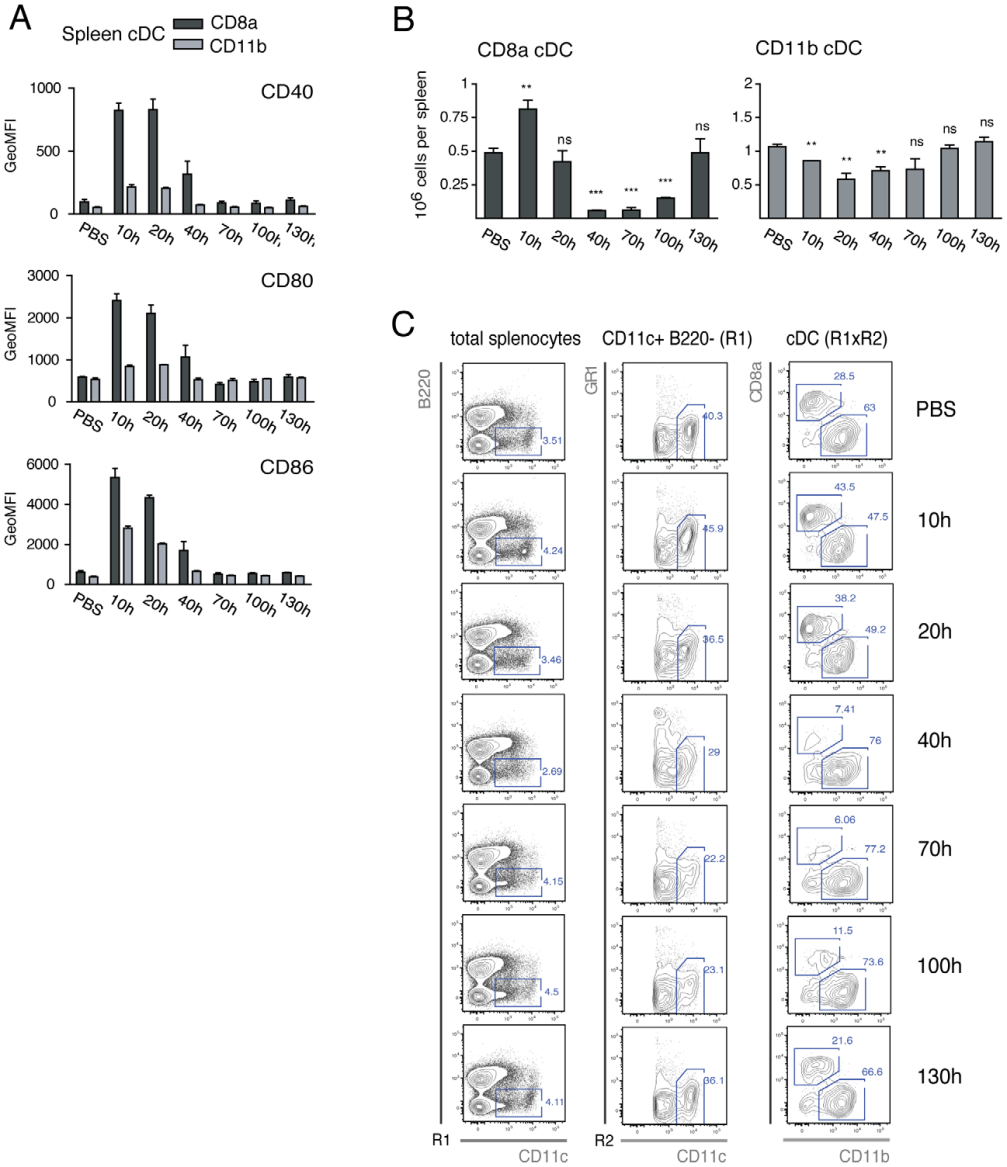
Kinetics of PolyIC-induced activation and loss of splenic cDC subsets. Mice were injected for several lengths of time with PolyIC (n = 3 per time-point) or PBS (time-points 0, 10, 70 or 130 h) as controls. **A**. The geometric mean of fluorescence intensity (GeoMFI) is shown for the activation markers CD40, CD80 and CD86 within each splenic cDC subset analyzed as in C. **B**. Total cells from each cDC subset per time-point as indicated. P-values indicate significance per time-point compared to injection with PBS. **C**. Gating strategy for the analysis of splenic cDC subsets, showing one representative sample. Splenic leukocytes were gated for CD11c B220^−^Gr-1^−^ cDC (R1xR2), and further segregated into CD8α or CD11b subsets. Data are representative of at least two independent experiments (except for time-point 130h).

Splenic cDC were analyzed at several time-points following injection of PolyIC in WT mice. In spleen, cDC are characterized as CD11c MHC-II B220^−^ and are commonly divided into the CD8α^+^ CD24^+^ CD11b^−^ SIRPα^−^ and CD8^−^ CD24^−^ CD11b^+^ SIRPα^+^ subsets with a steady-state ratio of 1∶3 [3]. The other major DC subset, the pDC, expresses intermediate levels of CD11c and is B220^+^. Moreover, infiltrating monocytes can differentiate into DC expressing CD11c and Gr-1 (moDC). We therefore identified splenic cDC as CD11c^+^ B220^−^ Gr-1^−^ and sub-divided them into CD8α or CD11b subsets. As expected, PolyIC induced cDC activation, characterized by up-regulation of the co-stimulatory molecules CD40, CD80 and CD86, with a peak between 10–20 h (**Fig. 1A**). Activation was more pronounced in the CD8α subset and correlated with a marked increase in their absolute numbers at 10 h (**Fig. 1B**). Between 40 and 100 h, however, the CD8α subset was dramatically reduced in numbers, with as little as 10% remaining when compared to starting numbers (**Fig. 1B and C**). In comparison, the CD11b subset showed a less pronounced activation as well as less expansion and subsequent reduction. Within 130 h of PolyIC treatment, both cDC subsets returned to steady-state levels. These results demonstrate that, in response to PolyIC injection, splenic cDC undergo transient activation followed by a marked reduction in the CD8α cDC subset *in vivo*.

### Splenic cDC depletion upon PolyIC stimulation is independent of both T cells and NK cells

**Figure 2:**
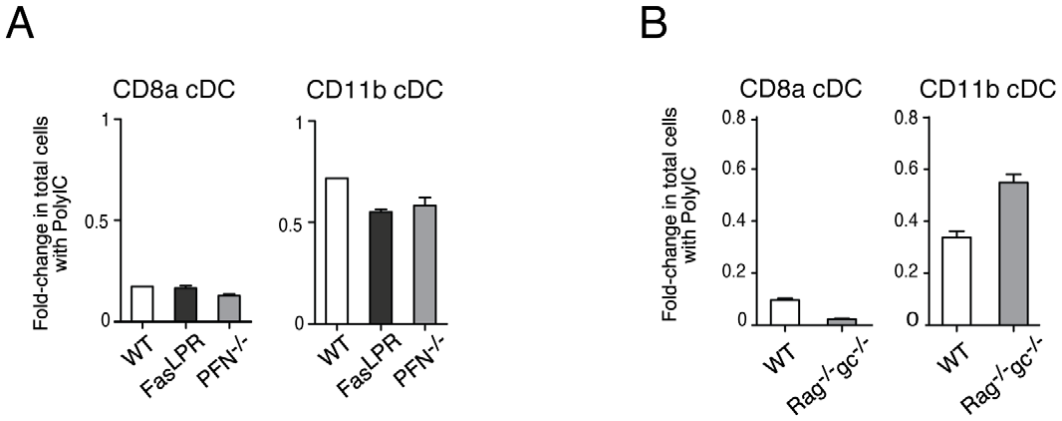
Loss of splenic cDC occurs independently of T, B or NK cells. **A**. *FAS^LPR^* or *perforin* ^−/−^ or control (WT) mice were treated with PBS (n = 3) or PolyIC (n = 3) and their splenic cDC analyzed after 40 h. The fold-change in total cells with PolyIC relative to PBS treatment is shown for each splenic cDC subset. **B**. *Rag2^−/−^γ_c_^−/−^* and control (WT) mice were injected with PBS (n = 3) or PolyIC (n = 3), and their splenic DC composition evaluated as described in Figure S1. The fold-change in total cells with PolyIC relative to PBS treatment is shown. Data are presented as mean +/− SD.

Previous reports have described the killing of DC by both CD4 and CD8 T cells, via Fas ligand and perforin, respectively [12], [13]. Splenic cDC depletion was therefore investigated in the *Fas^LPR^* spontaneous mutant (Fas-deficient) mice and perforin (*Pfn*)-deficient mice at 40 h of PolyIC treatment. In neither case was the loss of splenic cDC prevented (**Fig. 2A**), showing that the previously described CD4 and CD8 T cell controls of DC populations by these factors are not involved in PolyIC-induced splenic cDC depletion. To further evaluate the contribution of NK cells and/or T cells in this loss of splenic cDC, we injected PolyIC into mice lacking both cell types due to deficiency in Rag-2 and the cytokine receptor common γ-chain (γ_c_) (*Rag-2^−/−^γ_c_^−/−^*) (**Fig. 2B** and **Fig. S1A**). Treatment of *Rag-2^−/−^γ_c_^−/−^* mice with PolyIC induced a marked reduction in the numbers of splenic cDC (**Fig. S1B**). The fold-change in total cells with PolyIC showed that CD8α cDC were more extensively depleted, while CD11b cDC were less reduced, in *Rag2^−/−^γ_c_^−/−^* mice compared to WT mice (**Fig. 2B**). Interestingly, *Rag-2^−/−^γ_c_^−/−^* mice showed a steady-state CD8α-to-CD11b cDC ratio close to 0.8, in contrast to WT mice which display a ratio of ∼0.3. Such altered ratio is seen in all mice lacking B cells (K.S. and D.V., unpublished observations) and may have influenced the pattern of depletion upon PolyIC treatment in *Rag-2^−/−^γ_c_^−/−^*.

Regardless, these results demonstrate that a T cell- and NK cell-independent mechanism must reduce spleen cDC numbers and thereby limit DC responses to PolyIC stimulation.

### Depletion of splenic cDC also occurs upon treatment with different TLR-Ls and pathogenic infection

It has been shown that both CpG (TLR9-L) and LPS (TLR4-L) induce loss of splenic DC, albeit with limited distinction and quantitation of DC subsets [15]. We therefore examined whether preferential depletion of CD8α cDC was unique to PolyIC, or whether it could also be observed with other PAMPs. At 12 h, both CpG and LPS induced activation of the two cDC subsets (**Fig. S2A**), as well as a reduction in their cell numbers at 40 h (**Fig. S2B**). Interestingly, as in the case of PolyIC, LPS and CpG induced a greater activation and cDC loss in CD8α cDCs compared to CD11b cDCs. While CpG induced more modest effects, LPS treatment caused an extent of activation and subsequent depletion of CD8α cDCs comparable to PolyIC.

**Figure 3:**
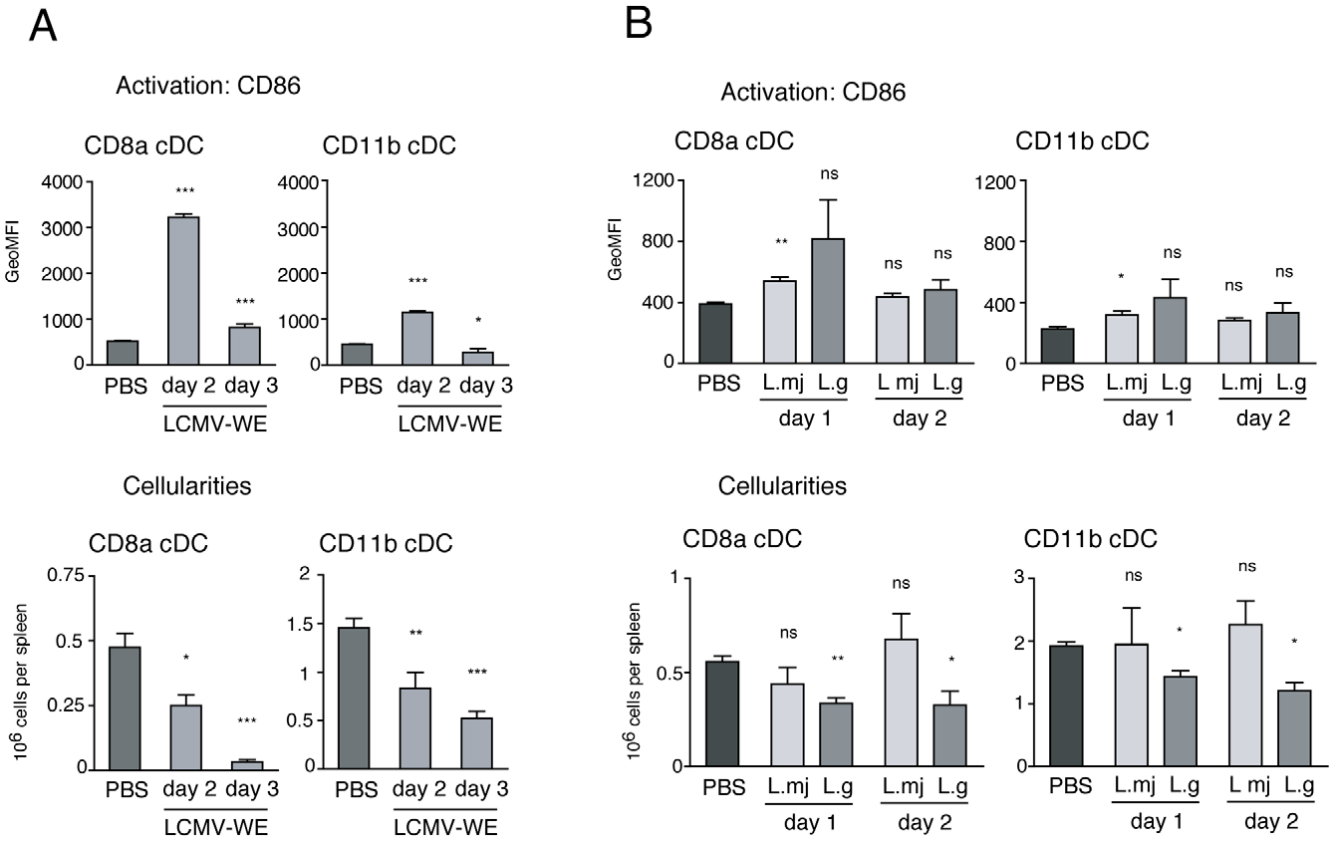
Splenic cDC depletion during infection with LCMV or *Leishmania*. **A**. Mice were treated with LCMV-WE or PBS (control). Splenic cDC activation (CD86 expression) and total cells were analyzed on day 2 (n = 3) and day 3 (n = 4) (control; n = 7). **B**. Mice were infected with *L. major* or *L. guyanensis*, or injected with PBS (control). Splenic cDC activation (CD86 expression) and total cells were analyzed on day 3 (n = 3) and day 6 (n = 3) (control; n = 4). Data are presented as mean +/− SD. P-values indicate significance per inoculation compared to injection with PBS.

PolyIC is a synthetic dsRNA analog that mimics genome or replication intermediates often found in viruses. In order to examine whether cDC depletion also occurs during infection with live pathogens *in vivo*, we analyzed the splenic cDC composition in mice at early time-points following challenge with LCMV or *Leishmania* parasites. Infection with 20′000 pfu of LCMV-Armstrong has previously been shown to reduce the numbers of both splenic cDC subsets by ∼50% at day 3 [16]. In our study, we inoculated mice i.v. with 10′000 pfu of LCMV-WE, a more aggressive and persistent strain of LCMV [17]. Early activation (CD86) on day 2 was accompanied by partial loss of cDC, and even more profound loss was observed on day 3 (**Fig. 3A**). Again, the CD8α cDC subset was more severely affected than the CD11b subset. Compared to the previously reported data on LCMV-Armstrong infection [16], the more aggressive LCMV-WE strain induced a bigger loss of cDC.

In addition, we challenged mice with the two *Leishmania* species, *L. major* and *L. guyanensis*, causing cutaneous and mucocutaneous leishmaniasis, respectively. *Leishmania* parasites stimulate surface as well as endosomal TLRs. Interestingly, *L. guyanensis* harbors a dsRNA virus that can provide additional TLR-3 stimulation [18], similar to PolyIC. Systemic infection of mice with *L. major* or *L. guyanensis* resulted in activation (CD86) of both cDC subsets on days 2 and 3 (**Fig. 3B**). Significant loss of splenic cDC was only observed with *L. guyanensis* but not *L. major*, presumably due to the stimulation by *leishmaniavirus* carried in *L. guyanensis* (additionalTLR3-L component).

Altogether, these data show that loss of splenic cDC was not restricted to stimulation with synthetic PolyIC but was also found in more patho-physiologically relevant infectious settings.

### PolyIC modulates expression of pro- and anti-apoptotic Bcl-2 family genes in splenic cDCs *in vivo*

Given the dramatic loss of splenic cDC upon PolyIC treatment, independently of other immune cells, we wanted to distinguish between emigration and cell death. For this purpose, we first analyzed pro- and anti-apoptotic Bcl-2 family members in cDC subsets purified 14 h after treatment *in vivo* with PolyIC, a time shortly before their depletion (**Fig. S3A**). Due to the low numbers of DCs that can be obtained and the clearance of apoptotic cells *in vivo*, we examined modulation of Bcl-2 family members at the mRNA level by quantitative real time (qRT)-PCR, a potential early apoptotic event (**Fig. S3B**). Amongst the pro-apoptotic BH3-only members analyzed, a strong induction of Bim and, albeit to a lesser extent, Puma could be consistently detected in both cDC subsets. Of note, CD8α cDCs expressed a higher steady-state level of Bim (three-fold) and Puma (two-fold) compared to the CD11b subset (PBS controls). While Noxa displayed inter-experimental variability in CD8α cDCs, being on average maintained, it was also up-regulated in CD11b cDCs. Interestingly, Bid expression was clearly down-regulated with PolyIC in CD8α cDCs, while CD11b cDCs expressed lower levels in steady-state (PBS) as compared to CD8α cDCs and maintained these levels after treatment. In the anti-apoptotic Bcl-2 family members analyzed, Bcl-xL was clearly increased in both subsets, being in CD8α cDCs more extensively up-regulated. Bcl-2 was maintained in CD11b cDCs and slightly down-regulated in CD8α cDCs, while PolyIC up-regulated Mcl-1, and A1 slightly, in both subsets.

These results show a change in the transcriptional program of pro- and anti-apoptotic Bcl-2 family members upon PolyIC treatment, with the strongest induction seen for Bim in both subsets.

### Multiple BH3-only proteins mediate PolyIC-induced apoptosis of splenic cDC, including but not solely involving Bim

**Figure 4:**
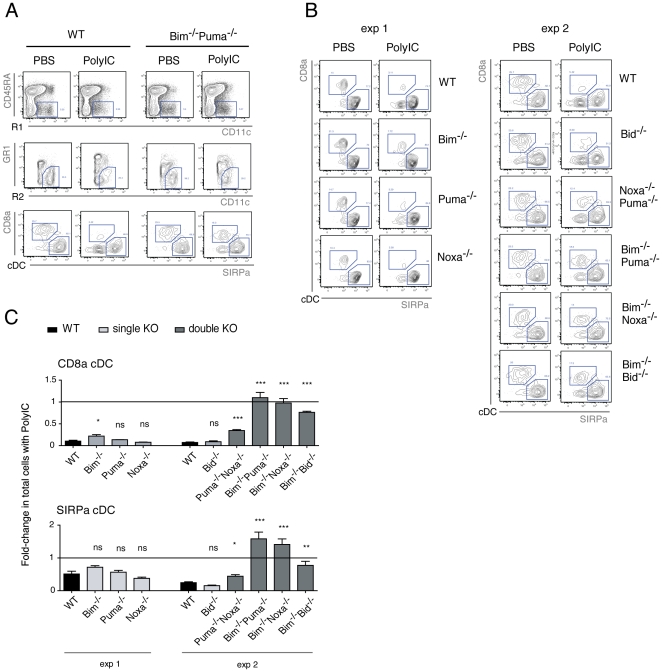
Loss of two BH3-only proteins, including Bim, protects cDC subsets from PolyIC-induced depletion. Mice of the genotypes indicated were injected with PolyIC (n = 2 or 3 per strain) or PBS (control; n = 2 per strain, except n = 1 for *Bid* ^−/−^) and splenic cDC numbers determined after 40 h. **A**. Strategy for the flow cytometry analysis of splenic cDC subsets. Total splenocytes were gated for CD11c CD45RA^−^ (R1), then CD11c^high^Gr-1^−^ (R2). In order to exclude splenic DC precursors, CD11b cDC were gated as CD8α^−^ SIRPα^high^ cells within the cDC (R1xR2), since fully developed CD11b cDC express high levels of Sirpα and intra-splenic cDC precursors express only intermediate levels of SIRPα and CD11c [64]. One representative example of a WT and a *Bim* ^−/−^ *Puma* ^−/−^ mouse injected with either PolyIC or PBS (control) is shown. **B**. Splenic cDC subset segregation as outlined in (A). One representative sample is shown per strain and treatment. **C**. The fold-change in total cells elicited by injection with PolyIC relative to PBS treatment is shown. Data are represented as mean +/− SD. P-values indicate significance per mouse strain as compared to WT controls.

Further to changes in gene expression, post-transcriptional and post-translational regulation of certain Bcl-2 family members is a common event, for instance proteolytic Bid activation. We wanted to examine whether the mitochondrial apoptotic pathway is responsible for splenic cDC depletion, and to identify the pro-apoptotic members possibly involved. To this end, we injected mice lacking Bim, Puma, Noxa, Bid or combinations of two of these BH3-only proteins (*Puma^−/−^Noxa^−/−^, Bim^−/−^Puma^−/−^, Bim^−/−^Noxa^−/−^ and Bim^−/−^Bid^−/−^*) with PolyIC and determined their cDC numbers after 40 h (**Fig. 4**). These different knock-out mice displayed normal DC populations (absolute numbers and ‘CD8α^+^: CD8α^−^ cDC’ ratio) in steady-state as compared to WT mice (**Fig. S4**). Following PolyIC treatment, mice singly deficient for Bid, Puma or Noxa displayed a reduction in both cDC subsets that was comparable to that seen in WT mice (**Fig. 4C**). Remarkably, while mice lacking Bim showed only a modest protection in cDC, similar to mice lacking both Puma and Noxa, a striking persistence of cDC numbers was seen in PolyIC-treated mice lacking Bim and a second BH3-only protein, either Bid, Puma or Noxa (**Fig. 4C**). This protection was already visually striking in the flow cytometric analysis of cDC subsets (**Fig. 4A and B**), which showed that the CD8α cDC subset in particular did not disappear after PolyIC injection, in contrast to their profound depletion observed in WT animals.

These results support that apoptosis is responsible for the depletion of splenic cDC subsets in response to PolyIC, and that the mechanism requires multiple BH3-only proteins, including Bim plus either Puma, Noxa or Bid.

### Type I IFN is necessary for the induction of splenic cDC depletion in response to PolyIC

**Figure 5:**
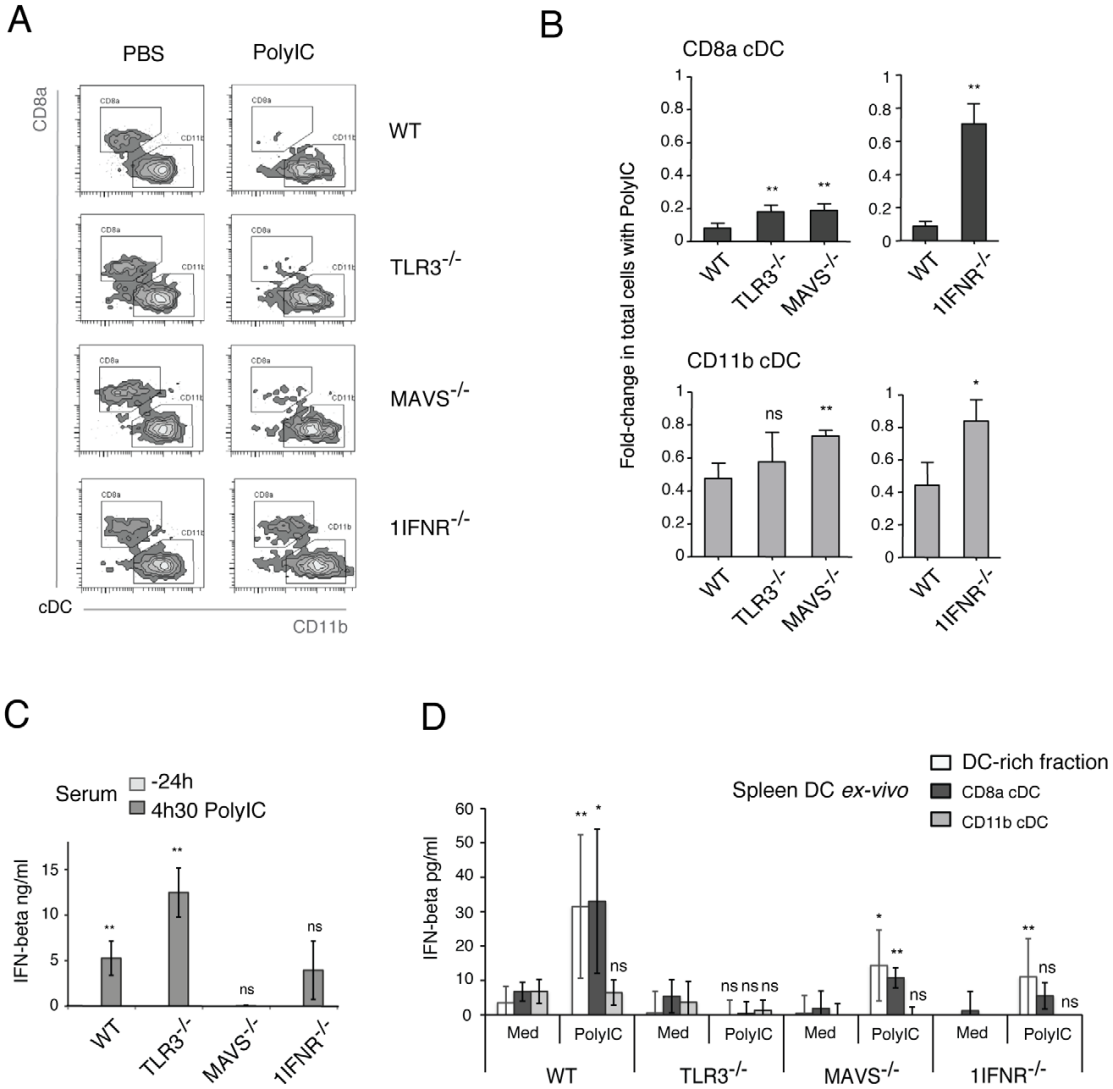
Type I IFN mediates splenic cDC loss and is cooperatively induced by TLR3 and MAVS *in vivo*. **A** and **B**. WT, *TLR*3^−/−^, *MAVS* ^−/−^ and *1IFNR* ^−/−^ mice were treated with PBS (control) or PolyIC. Splenic cDC were analyzed at 40 h as described before. The segregation of cDC into CD8α and CD11b subsets is shown (**A**) for one representative experiment per strain. The fold-change in total cells with PolyIC relative to PBS treatment is shown (**B**) for experiments with *TLR*3^−/−^ (n = 4) and *MAVS* ^−/−^ (n = 3) mice with WT mice as controls (n = 4), and for *1IFNR* ^−/−^ (n = 3) mice with WT mice as controls (n = 3). P-values indicate significance per mouse strain as compared to WT controls. **C**. WT, *TLR*3^−/−^, *MAVS* ^−/−^, and *1IFNR* ^−/−^ mice were injected with PolyIC and the levels of serum IFNβ examined 24 h before and 4.5 h after treatment (n = 3, representative of two independent experiments). P-values indicate significance comparing PolyIC treatment to PBS controls, per strain. **D**. Splenic DC fractions were isolated from WT, *TLR*3^−/−^, *MAVS* ^−/−^ and *1IFNR* ^−/−^ mice as depicted in Figure S6 and cultured in medium (control) or with PolyIC for 4 h. IFNβ production was analyzed in supernatants (n = 6 for DC-rich fractions, n = 5 for cDC subset fractions, from two independent experiments). P-values indicate significance comparing PolyIC treatment to medium alone, per strain. Data are presented as mean +/− SD.

We wanted to identify the signaling events downstream PolyIC detection that induced DC death. Double-stranded RNA can be detected by TLR3 in the endosome or by Rig-like Helicases (MDA-5 and RIG-I) that survey the cytosol compartment and signal via the adaptor MAVS. PolyIC in particular is a mimic of long dsRNA (>3 kbp), that may be detected by TLR3 and MDA-5, but not RIG-I [19]. CD8α cDC are known to express higher levels of TLR3 than the CD11b subset [4], [20] (**Fig. S5B**). The expression of MAVS was found to be similar amongst splenic cDC subsets in WT mice (**Fig. S5A**). Recent proteomic analysis of DC subsets has furthermore confirmed such selectively higher levels of TLR3 in CD8α cDC at the protein level [21]. This might explain the greater extent of activation, expansion and subsequent depletion seen in CD8α cDC compared to CD11b cDC. PolyIC-induced cDC loss was therefore investigated in mice lacking either *Ttlr3* or the adaptor *Mavs* (downstream of MDA-5) (**Fig. 5**). Surprisingly, loss of either *Tlr3* or *Mavs* only resulted in partially increased persistence of splenic cDC following PolyIC treatment (**Fig. 5A and B**). This suggested that apoptosis may be induced by converging factors downstream dsRNA detection, such as the induction of Type I IFNs. Accordingly, splenic cDC depletion following PolyIC administration was analyzed in mice lacking the alpha chain of the Type I IFN Receptor (*Ifnar1^−/−^*, hereafter termed *1IFNR^−/−^* mice)^1^. Remarkably, loss of *1IFNR* clearly protected both splenic cDC subsets from depletion following PolyIC treatment (**Fig. 5A and B**).

### TLR3 and MAVS cooperate in IFNß production in response to PolyIC treatment *in vivo*

The results showed that production of Type I IFN was the critical event that caused DC loss *in vivo* in response to PolyIC. Deficiency of either TLR3 or MAVS did not protect DC from PolyIC, suggesting that both TLR3 and MAVS cooperate in Type I IFN production. We therefore assessed the relative contributions of TLR3 and MAVS in IFNß production in response to PolyIC. First, systemic production was analyzed in sera of WT, *TLR3^−/−^*, *MAVS^−/−^* or *1IFNR^−/−^* mice after 4.5 h of PolyIC injection. In agreement with previous reports [6], [22], MAVS was required for systemic production of this cytokine (**Fig. 5C**). Secondly, we analyzed IFNß production by DC. Splenic DC represent a minor fraction of cells *in vivo* (e.g. CD8α cDC are 0.25% of total cells in spleen) and their relative contribution to IFNß production might not be detectable in serum or cultures of whole splenocytes. We therefore analyzed DC-rich spleen fractions (containing cDC subsets, pDC and B cells) as well as purified CD8α or CD11b subsets. These were obtained from WT, *TLR3^−/−^*, *MAVS^−/−^* or *1IFNR^−/−^* mice and cultured with PolyIC or medium alone for 4 h (**Fig. 5D** and **Fig. S6**). In WT samples, IFNß was induced in both the DC-rich fraction and the purified CD8α subset, whereas the CD11b subset showed no secretion (**Fig. 5D**). Importantly, loss of *TLR3* abrogated production of IFNß by CD8α cDC, while *MAVS^−/−^* CD8α DC showed impaired although still detectable induction of IFNß (**Fig. 5D**). These results support that TLR3 and MAVS cooperate to induce IFNß in response to PolyIC, in a cell-type specific manner. Isolated splenic CD8α cDC required TLR3, while other cells relied on MAVS and produced IFNß systemically. Presumably, the relatively small contribution of splenic CD8α cDC to systemic IFNß production is not detectable in the serum of MAVS^−/−^ mice.

### CD8α DC lines rely on TLR3 for IFNß production and apoptosis with PolyIC

In addition to IFNß production by DC, we wanted to validate that DC undergo apoptosis in response to PolyIC, in isolation from other cells (*in vitro*), and assess the requirement of TLR3. Of note, several technical difficulties render the study of DC apoptosis challenging, including the scarcity of DC in the mouse and the fact that dying cells are rapidly cleared *in vivo* (rendering their detection difficult). We initially assessed whether apoptosis of DC induced by PolyIC treatment *in vivo* could be detected by a subsequent enrichment and 4 h culture of DC *ex vivo*, allowing for phosphatidylserine translocation (AnnexinV staining) with minimal phagocytosis by surrounding cells (**Fig. S7A**). Although a slight increase in AnnexinV staining was seen in cDC from PolyIC-treated mice (**Fig. S7B**), the rapid spontaneous activation and apoptosis of splenic cDC *ex vivo* (4 h in cDC from PBS-treated, **Fig. S7B** and **C**) rendered analysis of PolyIC-induced apoptosis in splenic cDC extremely difficult.

In order to avoid spontaneous apoptosis of DC, we have taken advantage of novel CD8α DC lines derived from the culture of tumors arising in our recently reported transgenic mouse model of Langerhans cell hiostiocytosis [23], harbouring the SV-40LgT oncogene under the control of the DC-specific CD11c promoter (**Fig. S8A**). The DC lines derived share all tested features with freshly-isolated CD8α DC, such as PAMP-induced up-regulation of MHC class II and co-stimulation molecules, cytokine secretion upon activation, and antigen-presentation and cross-presentation *(Fuertes Marraco and Grosjean et al, manuscript in preparation).* Following activation with PolyIC (**Fig. S8B**), DC lines displayed induction of apoptosis as confirmed by the standard AnnexinV/7-AAD staining as well as mitochondrial depolarization (**Fig. S8C**). Pertinently, the kinetics of apoptosis in DC lines was reminiscent of the depletion of splenic cDC observed in response to PolyIC *in vivo* (**Fig. 1**).

**Figure 6:**
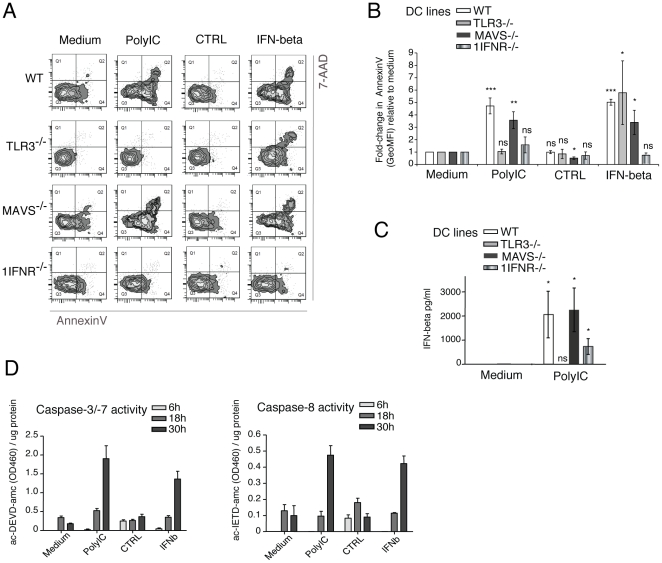
DC lines show TLR3-dependent IFNβ secretion with PolyIC and sufficiency of IFNβ for apoptosis induction. **A and B**. WT, *MAVS* ^−/−^, *TLR*3^−/−^ and *1IFNR* ^−/−^ DC lines were cultured in medium, PolyIC, CTRL supernatant or IFNβ-containing supernatant (n = 3, representative of at least three independent experiments). AnnexinV/7-AAD staining was performed at 48 h. One representative sample (A) and the fold-change in the GeoMFI of AnnexinV with treatment (B) are shown. **C**. Supernatants from WT, *TLR*3^−/−^, *MAVS* ^−/−^ and *1IFNR* ^−/−^ DC lines were assayed for IFNβ levels upon treatment with medium or PolyIC for 4 h (n = 3 independent experiments). **D**. WT DC lines were treated with medium, PolyIC, CTRL supernatant or IFNβ-containing supernatant (n = 3) for the time-points indicated and analyzed for caspase-3/-7 activity and caspase-8 activity. Data are presented as mean +/− SD. Where shown, P-values indicate significance comparing PolyIC treatment to medium alone, per strain.

Several genetically deficient DC lines were derived from tumors in CD11c:SV40LgT-TG mice crossed to various genetically deficient backgrounds, including *1IFNR^−/−^*, *TLR3^−/−^* and *MAVS^−/−^*. Upon treatment with PolyIC for 48 h, *1IFNR^−/−^* DC lines were protected from PolyIC-induced apoptosis (**Fig. 6A and B**), in agreement with the requirement for the 1IFNR for splenic cDC loss *in vivo* (**Fig. 5A and B**). Strikingly, *TLR3^−/−^* DC lines were also resistant to PolyIC, whereas *MAVS^−/−^* DC lines remained normally susceptible (**Fig. 6A and B**). Importantly, *MAVS^−/−^* DC lines produced IFNß in response to PolyIC, whereas the resistance of *TLR3^−/−^* DC lines correlated with a lack of IFNß production (**Fig. 6C**), in agreement with the requirement of TLR3 for IFNß production by freshly isolated splenic CD8α cDC (**Fig. 5D**).

### IFNβ is sufficient to induce apoptosis of DC lines *in vitro* and splenic cDC loss *in vivo*

We next investigated whether Type I IFN was not only necessary but also sufficient to induce apoptosis of splenic cDC. Notably, except for *1IFNR^−/−^* DC lines, all other DC lines readily underwent apoptosis after 48 h of treatment with IFNß, including the PolyIC-resistant *TLR3^−/−^* DC lines (**Fig. 6A and B**). In addition, effector caspase-3/-7 and initiator caspase-8 activities were clearly evident in WT DC lines after ∼30 h of stimulation with either PolyIC or IFNß (**Fig. 6D**).

**Figure 7:**
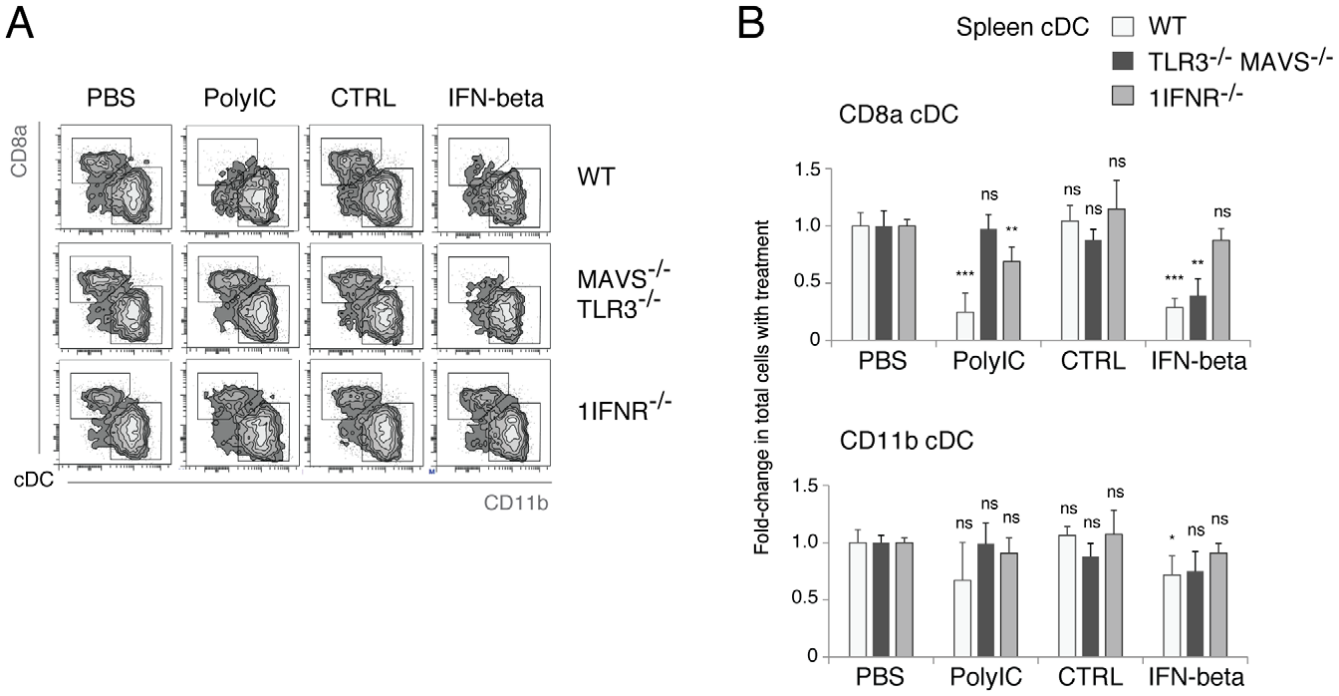
IFNβ is sufficient to induce splenic cDC loss *in vivo*. WT (n  =  4), *TLR3^−/−^ MAVS^−/−^* (n = 3) or *1IFNR* ^−/−^ (n = 4) mice were treated with PBS, PolyIC, CTRL cells or IFNβ-producing cells and the splenic cDC composition was analyzed after 40 h by flow cytometry as described in Figure 1B. **A**. The segregation of splenic cDC into CD8α+ or CD11b+ subsets is shown for one representative sample. **B**. The fold-change in total cells with treatment relative to PBS controls per cDC subset is shown. Data are presented as mean +/− SD, from two independent experiments. P-values indicate significance per treatment as compared to PBS controls, per mouse strain.

Further to the preliminary evidence in DC lines, it was critical to investigate the sufficiency of IFNß to induce loss of splenic cDC *in vivo*. Experiments using WT and *1IFNR^−/−^* mice showed that injection of IFNß was sufficient to deplete WT splenic cDC subsets to an extent similar to that achieved by treatment with PolyIC (**Fig. 7**). Moreover, *TLR3^−/−^MAVS^−/−^* double deficient mice were generated in order to test whether TLR3 and MAVS have a cooperative action in the loss of splenic cDC in response to PolyIC. Remarkably, *TLR3^−/−^MAVS^−/−^* splenic cDC were found to be resistant to PolyIC injection, but remained susceptible to treatment with IFNß (**Fig. 7**). These data support the co-operative action between TLR3 and MAVS in IFNß production *in vivo*, and demonstrate that IFNß is alone sufficient to induce splenic cDC loss *in vitro* and *in vivo*.

## Discussion

### PolyIC kills splenic cDC *in vivo*, in particular CD8α cDC, via the conjunction of multiple BH3-only proteins, including but not solely involving Bim

There are contradictory reports on the effects of microbial stimulation on DC viability, and to date no reports exist on apoptotic mechanisms controlling DC populations *in vivo*. One prediction is that recognition of pathogens would enhance DC survival to guarantee potent DC function, thus promoting efficient adaptive immune responses. Conversely, given the harmful consequences of exaggerated immune responses, strong microbial stimulation might be expected to create a need for increased DC turnover. We show here that splenic cDC undergo apoptosis in response to PolyIC, particularly affecting the CD8α subset. Furthermore, loss of splenic cDCs occurred in the absence of T cells or NK cells, pointing to an apoptotic mechanism that is intrinsic to DCs and distinct from previous reports [12], [13]. In our hands, concomitant CD40 cross-linking did not rescue CD8α DC from PolyIC-induced death (data not shown) [24].

Remarkably, while loss of Bim alone afforded only minor protection of DC, no DC loss was seen in PolyIC-injected mice lacking Bim plus either Puma, Noxa or Bid. Such correlation supports that DC apoptosis takes place *in vivo* in response to PolyIC. Whether DC that are deficient for two BH3-only proteins (including Bim) are generally more resistant to apoptosis cannot be excluded. To test the latter hypothesis, alternative unrelated treatments that induce apoptosis in splenic cDC would need to be identified and investigated in the different BH3-only deficient mice. Of note however, the different mice deficient for BH3-only members did not display increased DC populations in steady-state (neither numbers nor CD8α^+^-to-CD8α^−^ cDC ratio, **Fig. S4**). This excludes the presence of abnormally high starting numbers of splenic cDC in mice deficient for BH3-only members that would then render DC loss with PolyIC less significant. The involvement of Bim together with other BH3-only proteins in splenic cDC loss suggests that it is the overall balance between the pro-apoptotic BH3-only proteins and the anti-apoptotic Bcl-2 members, which regulates cDC lifespan after PolyIC stimulation. A similar overlapping action of several BH3-only proteins has been reported in neutrophil apoptosis and in the context of inflammatory arthritis [25], [26]. To our knowledge, this is the first report on the apoptotic mechanism regulating activated DC lifespan *in vivo*, mediated by the overlapping action of pro-apoptotic BH3-only proteins, including but not solely involving Bim.

### The CD8α cDC subset is predominantly lost

Intriguingly, in response to PolyIC, LPS and CpG, the CD8α cDC subset showed greater up-regulation of co-stimulatory markers (e.g. CD86) and also greater subsequent depletion than the CD11b subset. Notably, CD8α cDCs displayed a higher steady-state level of gene expression for Bim, Puma and Bid as compared to CD11b cDCs (PBS controls), supporting the hypothesis of a higher intrinsic susceptibility of CD8α cDCs to apoptosis. Clearly in both subsets, PolyIC treatment further up-regulated Bim and Puma. Interestingly, CD8α cDC also strongly up-regulated anti-apoptotic Bcl-xL in response to PolyIC, and down-regulated pro-apoptotic Bid to the levels of CD11b cDC, which were maintained upon treatment. This could reflect an attempt by CD8α cDC to overcome the initially higher pro-apoptotic to pro-survival Bcl-2 family member ratio than CD11b cDC, as a negative feedback mechanism in order to temporarily sustain their activation. The aforementioned results using mice genetically deficient for pro-apoptotic Bcl-2 family members nevertheless show that ultimately, in both subsets, Bim with a second BH3-only protein is sufficient to tilt the pro-to-anti-survival balance towards cell death. Possible speculations on such preferential death of the CD8α cDC subset are discussed further below.

### Type I IFN is necessary and sufficient for PolyIC-induced DC death, with a cell-type specific and cooperative involvement of TLR3 and MAVS *in vivo*

A fundamental question was to dissect the signaling events that induced apoptosis of DC, and not only their activation, in response to PolyIC treatment. We show that DC depletion required the 1IFNR, and IFNβ was necessary and sufficient to kill DC lines and deplete splenic cDC *in vivo*. In our hands, IFNß was predominantly detected over IFNα in DC culture supernatants (data not shown). However, while we addressed induction of DC apoptosis with IFNß, it cannot be excluded that other members of the Type I IFN family, notably the different IFNα proteins, may induce similar DC death.

Interestingly, neither endosomal TLR3 nor cytosolic MAVS were individually indispensable, rather double deficiency (*TLR3^−/−^ MAVS^−/−^*) was necessary to prevent DC loss *in vivo*. In response to PolyIC, TLR3/TRIF and MDA-5/MAVS signaling axes have been reported to cooperate in DC activation, antibody production, as well as CTL and NK responses [6], [22], [27]. In agreement with the latter reports [6], [22], systemic IFNβ production in response to PolyIC required MAVS. However, we have found that splenic cDC, in particular the CD8α subset and DC lines, rely on TLR3 for IFNβ production. Such TLR3 requirement in CD8α cDC is in contrast to a previous report using isolated DEC205+ (CD8α+) DC [6], where rather MAVS is required. To our knowledge, we provide first evidence that CD8α cDC rely on TLR3 for IFNß production and *in vitro* apoptosis in response to PolyIC, in line with their characteristic expression of TLR3 [20], [21]. Importantly, treatment with IFNβ alone was sufficient induce apoptosis in TLR3^−/−^ DC lines and loss of splenic cDC in mice doubly deficient for both TLR3 and MAVS.

Our findings support the hypothesis that both TLR3 and MAVS cooperate *in vivo* in a cell-type specific manner to produce IFNβ upon PolyIC treatment and consequently splenic cDC loss. However, different cell types display a differential requirement for either of these signaling pathways, with systemic IFNß production depending on MAVS, and splenic CD8α cDC (including DC lines) requiring TLR3. This furthermore implies that both autocrine and paracrine Type I IFN production induces DC death.

Type I IFNs are well recognized anti-viral factors; virtually all cells may respond to Type I IFN, inducing diverse, and ubiquitous or cell-type specific effects [28]. Due to the pleiotropic nature of Type I IFN effects, many of the details of Type I IFN mechanism of action and their particular relevant scenarios remain to be elucidated [29]. Inducing an antiviral state, Type I IFNs may cause growth arrest and apoptosis in several cell types, particularly in transformed cells. As part of their anti-viral effectiveness, Type I IFN also induce immune activation, and may further induce proliferation and sustained survival of certain immune cell types such as T cells [30].

Induction of Type I IFN by PRR has been recently shown to be critical for DC function, in particular in the context of the adjuvant effect of PolyIC [6]. There is however limited evidence concerning the impact of Type I IFN in DC viability. *In vitro*, Type I IFN can antagonize the survival effects of TLR-Ls in fibroblasts and human MoDC [31], and IFNβ induces death in BM-DC cultures only when they are concomitantly treated with a stimulatory cocktail [32]. Generally, Type I IFN alone is not sufficient to induce apoptosis in most cell types [29]. Rather, the pro-apoptotic effect of Type I IFN generally relies on sensitization to concomitant apoptotic triggers, such as treatments with TNF family members [33]. In addition, Type I IFN may itself induce TNF family members such as TRAIL, and consequently apoptosis [34], as shown in the context of multiple myeloma. TNFa is another important cytokine for DC activation, for example during viral infection [35]. Furthermore, the contribution of Bid in PolyIC-induced DC loss that we observed (**Fig. 4**) raises the question of whether death receptor signaling contributes to DC apoptosis. We therefore tested for a possible involvement of TNFa or TRAIL in splenic cDC depletion with PolyIC-induced Type I IFN. Reduction in both cDC subsets with PolyIC still occurred as extensively in *TNFa^−/−^* mice (**Fig. S9A**) or in WT mice treated with TRAIL-R2:Fc blockade (blocking soluble TRAIL) (**Fig. S9B**). Other genetic deficiencies within the TNF or TNF-R family, including *LTa^−/−^* or *TNFR1^−/−^TNFR2^−/−^* mice (data not shown), did not affect the loss of splenic cDC with PolyIC.

Our results show for the first time that IFNβ is sufficient to induce death of splenic cDC *in vivo*, in absence of accompanying stimuli, demonstrating a novel apoptotic effect of Type I IFN in a critical antigen-presenting cell.

### What may be the physiological significance of DC death upon PAMP/Type I IFN stimulation?

Given the critical function of DC for the induction of adaptive immune responses, the apoptosis of DC in response to a microbial-like stimulus and/or Type I IFN (critical anti-viral mediators), affecting in particular the cross-presenting CD8α cDC subset, may appear paradoxical. Several speculations may be advanced concerning the physiological significance that such DC death may have.

DC apoptosis may limit the spread of infection and allow for an adequate shutdown of immune responses. Critically, DC apoptosis must maintain a healthy balance between excessive DC clearance leading to immunosuppression and the maintenance of immune competence without causing autoimmunity. Aberrant accumulation of DC results in chronic lymphocyte activation and systemic autoimmune manifestations, as shown in transgenic mice expressing the baculoviral pan-caspase inhibitor p35 under the DC-specific CD11c promoter [9]. Similarly, Bim-deficient DC display a decrease in spontaneous death in culture and their adoptive transfer induces autoantibody production *in vivo* [14]. Conversely, a reduction in DC numbers has been linked to states of immune suppression, such as sepsis [36], [37] and chronic viral infection. Impairment of DC function during LCMV infection was reported and linked to immunosuppressive states, with the LCMV-WE being more immunosuppressive and persistent than LCMV-Armstrong [17]. Interestingly, we observed that LCMV-WE infection causes more extensive cDC depletion than previously reported for LCMV-Armstrong [16]. Several studies have detected an impairment of DC function upon TLR stimulation [38], similarly to chronic inflammatory or infectious states [39], [40]. This has been thought to be a consequence of loss of antigen uptake and processing in activated DC, but may well (at least in part) have been due to the here-described DC death.

1IFNR*^−/−^* mice succumb to infection with many viruses, showing the importance of Type I IFN for antiviral responses, including stimulation and clonal expansion of CTL [41], [42]. However, a Type I IFN response may also be detrimental under certain circumstances, such as infection with *listeria monocytogenes* [29], [43]. While Type I IFN is required for the initiation of adequate anti-viral responses, the DC apoptosis we report could be a negative secondary effect of Type I IFN, either when “inappropriately” induced or upon persistent infections. In support of this idea, persistence of viral infection and immune suppression has been correlated with reduced DC numbers and sustained levels of Type I IFN, in models such as LCMV-Armstrong infection in mice [44], as well as infections with HIV-1 in humans [45] and SIV in monkeys [46]. Collectively, our findings suggest that one major cause of transient or chronic immunosuppresion during infectious conditions is a consequence of apoptotic depletion of DC linked to sustained and/or aberrantly high Type I IFN levels.

The DC apoptosis we report is particularly relevant for the therapeutic exploitation of TLR-Ls and Type I IFNs (e.g. adjuvants, multiple sclerosis, cancer, Langerhans cell histiocytosis) [2], [47], [48], [49]. Both the adjuvant immune stimulatory and direct anti-proliferative properties of Type I IFN have a strong potential for therapeutic exploitation for cancer treatments [50], [51]. Similarly, IFNß has been used for the treatment of autoimmune diseases such as multiple sclerosis [52]. It would be critical to investigate whether current or developing therapies featuring the use of Type I IFN or TLR-Ls induce DC loss. Accordingly, therapeutic regimes should be carefully designed (e.g. dosage, timing of antigen/adjuvant treatment) in order to avoid the potentially deleterious side effects of DC apoptosis leading to immunosuppression.

CD8α DC are particularly potent at phagocytosing dying cells for antigen cross-presentation and at inducing CTL responses [53], being thus one key target for vaccines against cancer and infectious diseases [54], [55], [56]. Strategies for vaccine enhancement include the targeting of antigens to CD8α cDC [2], [57], for which PolyIC has been shown to be an optimal adjuvant [2]. Pertinently, several lines of evidence support that TLR3 expression by CD8α DC and downstream Type I IFN production is critical for the adjuvant effect of dsRNA (PolyIC), including cell-associated dsRNA for cross-presentation [58], [59], [60], [61]. Given the unique functions of CD8α cDC, their preferential death with a virus-like stimulus (PolyIC) and Type I IFN (anti-viral mediator), as compared to the other CD11b DC subset, appears contradictory. However, on the one hand, apoptosis may be an evolutionary adaptation to limit the spread of intra-cellular pathogens within such critical DC subset. On the other hand, apoptosis may serve as an efficient mechanism to amplify the response to intracellular pathogens, by an apoptosis-mediated “DC to DC cross-presentation” of antigens. Via this mechanism, infected cells (DC) would be sacrificed, while pathogen would be continuously processed and diluted amongst neighboring cross-presenting DC, correlating with pathogen load. Such apoptosis-driven “DC to DC cross-presentation” would be particularly relevant when antigen is limiting, or considering the naturally low frequency of DC, to mount an adequate and rapid adaptive immune response.

### Perspectives

Future experiments should shed light on the physiological impact of DC death with PolyIC and Type I IFN. It will be addressed whether DC death promotes amplification of cross-presentation, protects from autoimmune pathology, induces side-effect immunosuppression, and/or affects vaccine efficiency. In addition to antigen targeting to DC and the use of TLR-Ls as adjuvants, efforts to optimize vaccine efficacy also include increasing DC viability after activation [62], [63]. Our findings have the potential to contribute to the optimization of vaccine therapies, providing an important basis for the design of strategies to enhance DC viability and consequently function.

## Materials and Methods

### Ethics statement

Animal experiments were performed in strict accordance to the Swiss Federal Regulations. The protocol was approved by the “Service de la consommation et des affaires vétérinaires du Canton de Vaud”, Switzerland (Permit Number: 1847.1). All efforts were made to minimize suffering and minimize the number of mice needed to assess statistical significance and experimental reproducibility. The generation and use of mouse DC lines for *in vitro* research represents a considerable advance towards the implementation of the 3R (Refine, Reduce, Replace) principle in animal experimentation.

### Mice and treatments

The mice used were females or males aged at least 8 weeks. Mice were WT (C57BL/6) or Fas^LPR^, Perforin (pfn)^−/−^, Bim^−/−^, Puma^−/−^, Noxa^−/−^, Bid^−/−^, Puma^−/−^Noxa^−/−^, Bim^−/−^Puma^−/−^, Bim^−/−^Noxa^−/−^, Bim^−/−^Bid^−/−^, Rag2^−/−^γc^−/−^, Tlr3^−/−^, Mavs^−/−^, Tlr3^−/−^Mavs^−/−^, Ifnar1^−/−^, Tnfa^−/−^ (see strain nomenclature, genetic background and references in Table S1). Mice were injected intra-peritoneally (i.p.) with 50 ug PolyIC (Poly(I:C)-HMW from Invivogen) or, as a control, with PBS. No transfecting reagent or liposomal vehicle was used along with PolyIC. Infection with LCMV-WE consisted of intra-venous (i.v.) inoculation with 10′000 pfu. Systemic infection with Leishmania was achieved by injection of 20×10^6^ parasites i.v. and 3×10^6^ parasites in both hind foot-pads, using infectious stationary phase promastigotes of either L. major (LV39, MRHO/SU/59/P) or *L. (V.) guyanensis *(WHI/BR/78/M5313) strains. Treatment with IFNß in vivo was performed as described below.

### Flow cytometry-based quantitation of splenic cDC subsets

Splenocyte suspensions were obtained by digestion with collagenase D (1 mg/mL) and DNAse I (40 ug/mL) in 3% FCS-RPMI-1640 at 25°C for 30 min. Cell suspensions were passed through 40 um sieves and washed in PBS containing 5 mM EDTA and 5 ug/mL DNAse I and then resuspended in PBS containing 5 mM EDTA and 3% FCS. Total numbers of live splenic leukocytes were determined by staining with Trypan blue and counting under a microscope or by using the Casy® cell counter and analysis system. Fluorochrome-conjugated monoclonal antibodies for flow cytometry were either generated and conjugated in house or purchased from eBioscience and BioLegends. Antibodies were specific to CD11c (clone N418, PECy7, in house or eBioscience), CD45R-B220 (clone RA3-6B2, PECy5.5, eBioscience), CD45RA (clone 14.8, APC, in house), GR1/Ly6G (clone RB6-8C5, PE, BioLegends, or biotinylated/SAVPerCPPECy5.5, in house), CD8α (clone 54-6.7, FITC, eBioscience, or APC-Cy7, in house), CD11b (clone M1/70, APC, eBioscience), SIRPα (a.k.a. CD172, clone p84, FITC, in house), CD40 (clone 1C10, APC, eBioscience), CD80 (clone 16-10A1, PECy5, eBioscience) and CD86 (clone GL1, APC, eBioscience). Analyses of *Rag2^−/−^γc^−/−^* splenocytes included antibodies specific for CD3e (clone 145-2C11, PE, eBioscience), CD19 (clone 6D5, PECy5.5, eBioscience), and CD49b (clone pan-NK DX5, FITC, eBioscience). Analysis was performed on Canto or LSR II machines (Becton Dickinson) using FACSDiva and FlowJo (Becton Dickinson) for data processing. Total numbers of cDC subsets were based on the total splenic leukocyte count and the fractions of cells in each gate used to arrive at a given cDC subset.

### DC isolation, cultures and treatments

The medium composition was IMDM-glutamax (GIBCO 31980) supplemented with 8–10% heat-inactivated FCS, 10 mM Hepes, 50 uM b-mercaptoethanol, and 50 U/mL of penicillin and 50 µg/mL streptomycin. Cells were cultured at 37°C with 5% CO_2_. Different DC populations were obtained from spleens as depicted in **Fig. S6**. Briefly, splenocyte suspensions were fractionated by density centrifugation in isohexol carbohydrate medium (Nycodenz, Axis-Shield, Norway) at 1.077 g/cm^3^ [64]. The DC-enriched, light density fraction was collected as the ‘DC-rich fraction’ (**Fig. S6**, fraction 1). Purified cDC subsets (>95%) were obtained from DC-rich fractions using anti-CD11c antibody-coupled magnetic micro-beads (Miltenyi Biotech) and flow cytometry cell sorting of CD11c^+^/B220^−^ cells into CD8α DC or CD11b DC (**Fig. S6**, fraction 2 or 3, respectively) using a FACSAria cell sorter (Becton Dickinson). DC lines were derived as detailed in Materials and Methods S1. For apoptosis assays, DC lines were seeded at 2×10^5^ cells/mL the day prior and treated with medium, PolyIC at 5 µg/mL, or CTRL and IFNβ-containing supernatants as detailed below. For IFNß production assays, supernatants were obtained from DC cultures (DC lines or DC *ex vivo*) at 10^6^ cells/mL treated with medium or PolyIC at 25 ng/mL for 4 h. The PolyIC used was Poly(I:C)-HMW from Invivogen and no transfecting reagent or liposomal vehicle was used.

### Recombinant IFNß production and treatments

Recombinant murine IFNß was produced in house by lentiviral transduction of 1IFNR^−/−^ DC lines, allowing to obtain a DC-conditioned medium containing IFNß, in the absence of secondary effects from the IFNß on the producer cells (see Materials and Methods S1 for the details). Concentrated IFNß (1–5 ug/mL) was obtained using high cell density culture systems (CELLine AD1000, Integra-Biosciences) and diluted to 25 ng/mL of IFNß for experiments *in vitro*, with mock-transduced DC line supernatant used as a control. For treatments *in vivo*, mice were injected i.p. with 5×10^6^ IFNß-producing cells or mock-transduced cells (CTRL).

### IFNβ quantitation

IFNβ quantitation was performed on cell culture supernatants or peripheral blood sera by ELISA (PBL 42400, PBL Interferon-source).

### Apoptosis assays

#### AnnexinV/7-AAD

AnnexinV staining was performed using fresh AnnexinV-binding buffer (ABB) (14 mM NaCl, 2.5 mM CaCl2, 10 mM Hepes) containing AnnexinV-PE (BD Pharmingen) and 7-AAD (eBioscience), both at 1∶50, for 15 min at RT in the dark. All samples were analyzed within 30 min.

#### Caspase activity assays

The fluorogenic substrates used were ac-IETD-amc for Caspase-8 and ac-DEVD-amc for Caspase-3/-7 (Enzo Life Sciences). Caspase-activity assays were performed on DC line samples according to the manufacturer's protocol, measuring caspase-3/-7 activity after 60 min and caspase-8 activity after 120 min and normalizing data to protein content quantitated by the standard Bradford reaction.

### Statistical analyses

P-values were obtained using two-tailed unpaired t-tests with 95% confidence intervals (ns  =  not significant; *  =  p<0.05; **  =  p<0.01; ***  =  p<0.001).

## Supporting Information

Figure S1 **Flow cytometry analysis of Rag2^-/-^γ_c_^-/-^ mice treated with PBS or PolyIC.** *Rag2* ^-/-^ *γ_c_* ^-/-^ mice were injected i.p. with 50 ug PolyIC (n = 3) or, as a control, PBS (n = 3), and their splenic composition compared to that of similarly treated WT mice (n = 3 for PolyIC, n = 3 for PBS). Consistent with previous observations [65], flow cytometric analyses of the spleens of these rag2^-/-^γ_c_^-/-^ mice was complicated by the presence of auto-fluorescent cells. **A**. *Rag2* ^-/-^γ_c_^-/-^ mice lack B cells, T cells and NK cells. The autofluorescent cell population in the staining for B cells, T cells and NK cells is visible on a fluorochrome-empty flow cytometry channel (FL4). Staining profiles for representative PBS-treated mice is shown for each strain. **B**. Gating strategy for the analysis of splenic cDC, excluding pDC, monocyte-derived DC and autofluorescent cells by a first gate on GR1 and B220 double-negative cells (an additional flow cytometry channel was not available in the staining combination for cDC analysis). CD11c^+^ cells from the GR1^−^B220^−^ were selected for segregation into CD8a^+^ vs CD11b^+^ subsets. Data from one representative mouse for each strain and treatment is shown. **C**. Total numbers of each cDC subset were calculated on the basis of total splenocytes harvested multiplied by the fraction of cells per gate as indicated in (b). To obtain the fold-changes in the cDC subset cellularities elicited by injection with PolyIC, the total number of DC in the spleens of mice treated with PolyIC were divided by the total number of DC in the spleens of PBS-treated mice. P-values indicate significance per strain as compared to WT controls.
(TIF)

Figure S2 **Loss of splenic cDC after CpG and LPS injection.** Mice (C57BL6) were injected i.p. with PBS (control; n = 4), 50 ug of CpG (n = 3), 10 ug of LPS (n = 3) or, as comparison to data shown in Figure 1, with 50 ug PolyIC (n = 1). Splenic cDC subsets were analyzed at 12 h post-injection for expression of the activation marker CD86 (GeoMFI  =  geometric mean of fluorescence intensity) and at 40 h for total numbers of cDC subsets. P-values indicate significance per treatment compared to injection with PBS.
(TIF)

Figure S3 **Changes in gene expression of Bcl-2 family members in cDC treated with PolyIC** ***in vivo*** **.** Mice were injected with 50 ug PolyIC (n = 9) or PBS (control; n = 9). After 14 h, the spleens were harvested and pooled for cDC subset isolation and gene expression analysis. The experiment was performed twice in two independent laboratories as described in the Methods section, with a total of four biological replicates per sample. **a**. Strategy for flow cytometric analysis, gating and isolation of CD8a or CD11b cDC subsets from total splenocytes. One representative experiment is shown. **b**. Analysis of the levels of mRNA for pro-apoptotic Bcl-2 family members Bim (n = 4), Puma (n = 4), Noxa (n = 4), Bid (n = 3) and anti-apoptotic Bcl-2 (n = 4), Mcl-1 (n = 4), A1 (n = 3) and Bcl-xL (n = 3). Open symbols (○, ◊) correspond to qRT-PCR data generated at the WEHI (Melbourne), normalized to β-actin as a house-keeping gene. Closed symbols (•, ♦) correspond to qRT-PCR data generated at the DB-UNIL (Lausanne), normalized to TBP as a house-keeping gene. The primers used were as detailed in Table S2A. In order to obtain an inter-experimental and inter-laboratory comparison, a second normalization was performed relative to CD8a cDCs treated with PBS.
(TIF)

Figure S4 **Steady-state splenic cDC populations in mice deficient for BH3-only members.** Splenic cDC subsets were quantitated as described before amongst WT (n = 6), *Bim^-/-^* (n = 4), *Puma^-/-^* (n = 2), *Noxa^-/-^* (n = 4), *Bid^-/-^* (n = 3), *Puma^-/-^Noxa^-/-^* (n = 2), *Bim^-/-^Puma^-/-^* (n = 2), *Bim^-/-^Noxa^-/-^* (n = 4), *Bim^-/-^Bid^-/-^* (n = 2). **A**. Ratio of CD8a^+^-to-CD8a^−^ cDC per mouse strain as indicated. **B**. Total numbers of each cDC subset per spleen per mouse strain as indicated. Data are represented as mean +/- SD. P-values indicate significance per strain as compared to WT controls.
(TIF)

Figure S5 **Expression levels for** ***TLR*** **3,** ***MAVS*** **and the** ***IFNaR1*** **in splenic cDC subsets and DC lines.** Purified splenic CD8α as well as CD11b cDC subsets and DC line samples (n = 2 per sample) were examined for the levels of mRNA for *Mavs* and *Ifnar1* by qRT-PCR using the primers detailed in Table S2B. *Tlr3* mRNA expression was analyzed by semi-quantitative PCR.
(TIF)

Figure S6 **Strategy and flow cytometry analysis for the purification of DC-rich and cDC subset fractions.** Splenocyte suspensions were fractionated by density centrifugation in isohexol carbohydrate medium (Nycodenz, Axis-Shield, Norway) at 1.077g/cm3. The light density fraction was collected as the DC-rich fraction (fraction 1, as indicated). CD11c+ cDC were isolated from DC-rich fractions using anti-CD11c antibody coupled magnetic micro-beads (Miltenyi Biotech). Purified cDC subsets were obtained from cDC by flow cytometry cell sorting separating CD11c^+^/B220^−^ cells into CD8a or CD11b subsets, collecting fractions 2 and 3, respetively, as indicated. One representative sample is shown. The cDC represent 1-2% of total splenic leukocytes. Within the cDC population, typically ∼20% were CD8a^+^ cDC and ∼60% CD11b cDC. At least 5 mice were required to isolate ∼0.5×10^6^ CD8a^+^ cDC. Density centrifugation allowed for a ∼10-fold enrichment in DC prior to immunomagnetic bead selection. Enriched DC preparations subjected to flow-cytometric cell sorting contained >75% cDC, and resulting cDC subsets were purified to at least 95%.
(TIF)

Figure S7 **Activation and apoptosis in splenic cDC ex-vivo, isolated from either PBS- or PolyIC-treated mice.** **A**. Experimental outline. Briefly, WT mice (n = 2) were treated with PBS or PolyIC for 24h. Splenic cDC were enriched by density centrifugation and analyzed inmediately (“0h”) or after 4h of culture (“4h”). Analysis included the segregation of cDC subsets as before and the apoptosis staining AnnexinV (**B**) or the activation marker CD86 (**C**). Data are represented as mean +/- SEM.
(TIF)

Figure S8 **PolyIC induces activation and apoptosis in DC lines.** **A**. Phenotype of DC lines derived from CD11c:SV40LgT-transgenic mice (NB. harbouring the eGFP reporter for the transgene) [23], analyzed by flow cytometry and compared to splenic cDC in WT mice as indicated. **B and C**. DC lines (WT) were treated *in vitro* with 8 ug/mL poly I:C. **B**. Histograms showing the up-regulation of co-stimulatory markers CD40, CD80 and CD86 at 24 h of treatment with PolyIC (dark grey) as compared to unteated controls (pale grey). **C**. The kinetics of poly I:C treatment are shown for a representative experiment, with flow cytometry analysis of FSC vs SSC plots, AnnexinV/7-AAD double-staining and TMRM histograms at different time points as indicated.
(TIF)

Figure S9 **Splenic cDC loss with PolyIC still occurs in TNFa-/- mice or with TRAIL-R2:Fc blockade.** **A**. WT and *TNFa* ^-/-^ mice were treated with PBS (control) or PolyIC and splenic cDC were analyzed at 40 h as described before (n = 2 per treatment per mouse strain). The fold-change in total cDC subset cells with PolyIC relative to PBS treatment is shown per strain as indicated. **B**. WT mice were treated with PBS or TRAIL-R2:Fc 6h before and at 18h after treatment with either PBS or PolyIC (time 0h) and splenic cDC were analyzed at 40 h as described before (n = 2 per treatment). Total numbers of each cDC subset per are shown per treatment as indicated. Data are presented as mean +/- SD.
(TIF)

Materials and Methods S1 **Materials and Methods used for the experiments shown in Supporting Figures and related to Materials and Methods.**
(DOC)

Table S1 **Mouse strains used (related to Materials and Methods and Materials and Methods S1).**
(DOC)

Table S2 **Primers used for gene expression analyses (related to Materials and Methods S1).**
(DOC)
