# Supplementary figures and images for: Type I Interferon Drives Dendritic Cell Apoptosis via Multiple BH3-Only Proteins following Activation by PolyIC In Vivo
Source: PLoS One. 2011 Jun 2;6(6):e20189. doi: 10.1371/journal.pone.0020189 (PMC3107228; doi:10.1371/journal.pone.0020189)

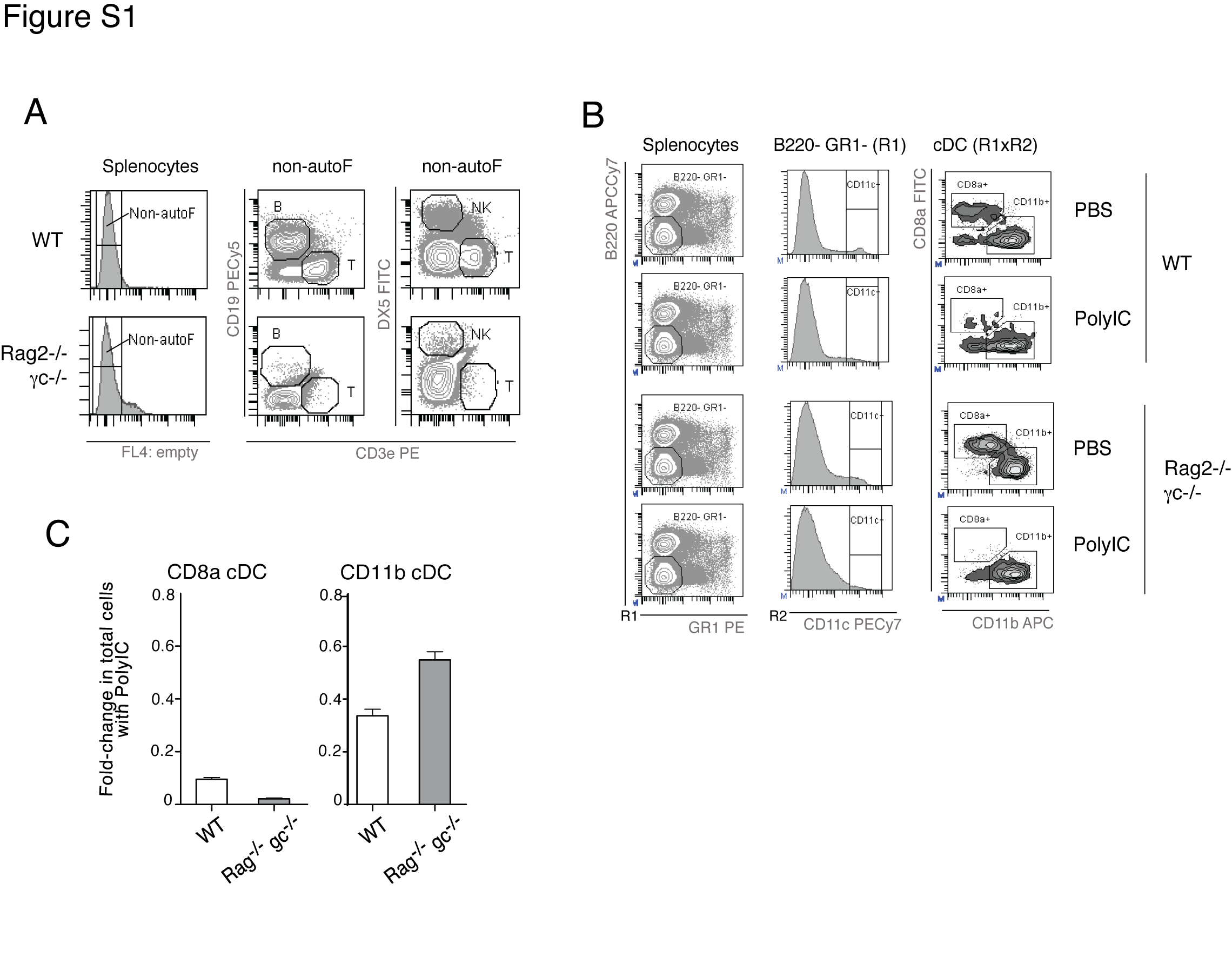

Supplement: Figure S1 — Flow cytometry analysis of Rag2-/-γc-/- mice treated with PBS or PolyIC. Rag2 -/- γc -/- mice were injected i.p. with 50 ug PolyIC (n = 3) or, as a control, PBS (n = 3), and their splenic composition compared to that of similarly treated WT mice (n = 3 for PolyIC, n = 3 for PBS). Consistent with previous observations [65], flow cytometric analyses of the spleens of these rag2-/-γc -/- mice was complicated by the presence of auto-fluorescent cells. A. Rag2 -/-γc -/- mice lack B cells, T cells and NK cells. The autofluorescent cell population in the staining for B cells, T cells and NK cells is visible on a fluorochrome-empty flow cytometry channel (FL4). Staining profiles for representative PBS-treated mice is shown for each strain. B. Gating strategy for the analysis of splenic cDC, excluding pDC, monocyte-derived DC and autofluorescent cells by a first gate on GR1 and B220 double-negative cells (an additional flow cytometry channel was not available in the staining combination for cDC analysis). CD11c+ cells from the GR1−B220− were selected for segregation into CD8a+ vs CD11b+ subsets. Data from one representative mouse for each strain and treatment is shown. C. Total numbers of each cDC subset were calculated on the basis of total splenocytes harvested multiplied by the fraction of cells per gate as indicated in (b). To obtain the fold-changes in the cDC subset cellularities elicited by injection with PolyIC, the total number of DC in the spleens of mice treated with PolyIC were divided by the total number of DC in the spleens of PBS-treated mice. P-values indicate significance per strain as compared to WT controls. (TIF) [file pone.0020189.s001.tif]

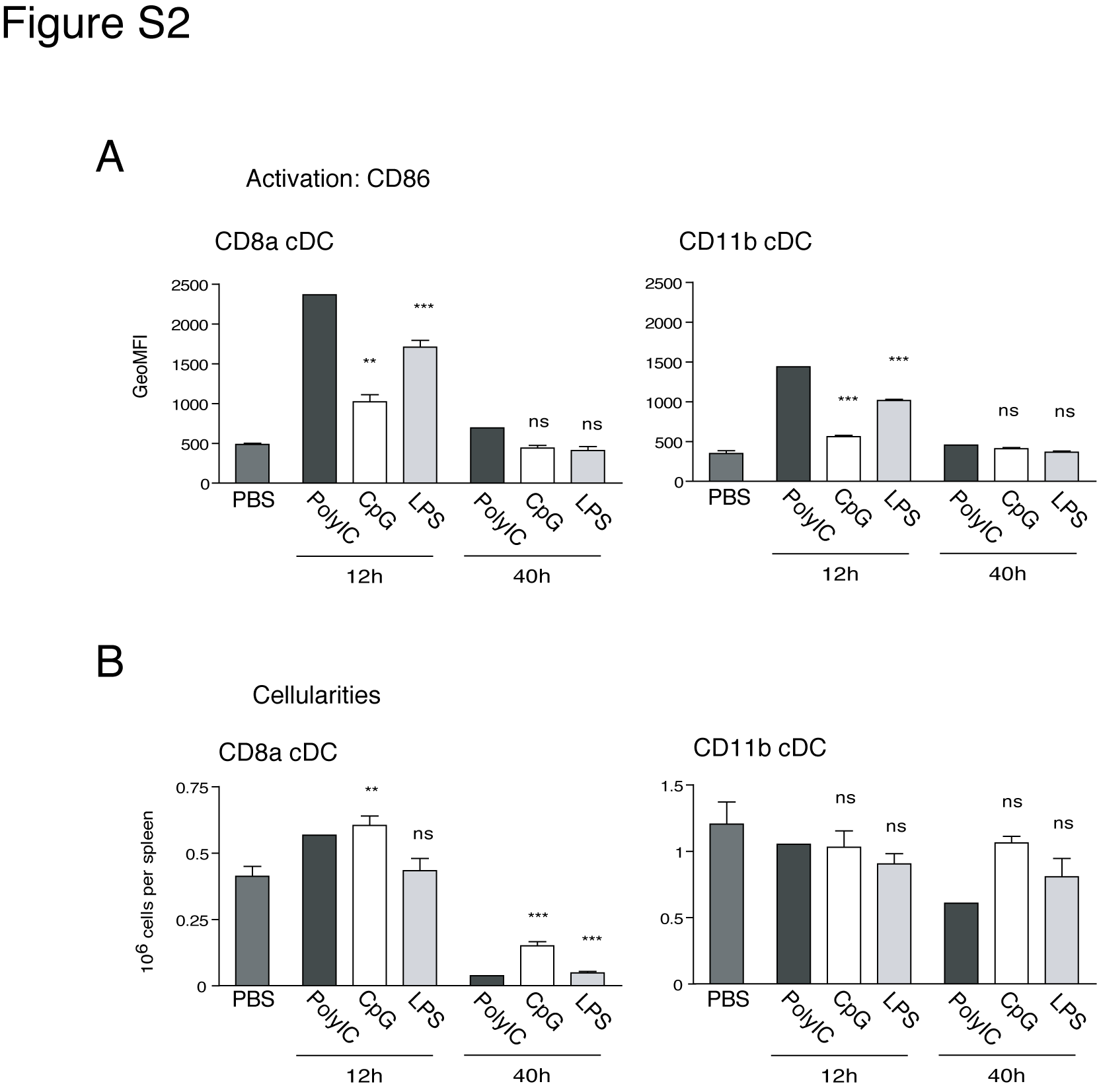

Supplement: Figure S2 — Loss of splenic cDC after CpG and LPS injection. Mice (C57BL6) were injected i.p. with PBS (control; n = 4), 50 ug of CpG (n = 3), 10 ug of LPS (n = 3) or, as comparison to data shown in Figure 1, with 50 ug PolyIC (n = 1). Splenic cDC subsets were analyzed at 12 h post-injection for expression of the activation marker CD86 (GeoMFI = geometric mean of fluorescence intensity) and at 40 h for total numbers of cDC subsets. P-values indicate significance per treatment compared to injection with PBS. (TIF) [file pone.0020189.s002.tif]

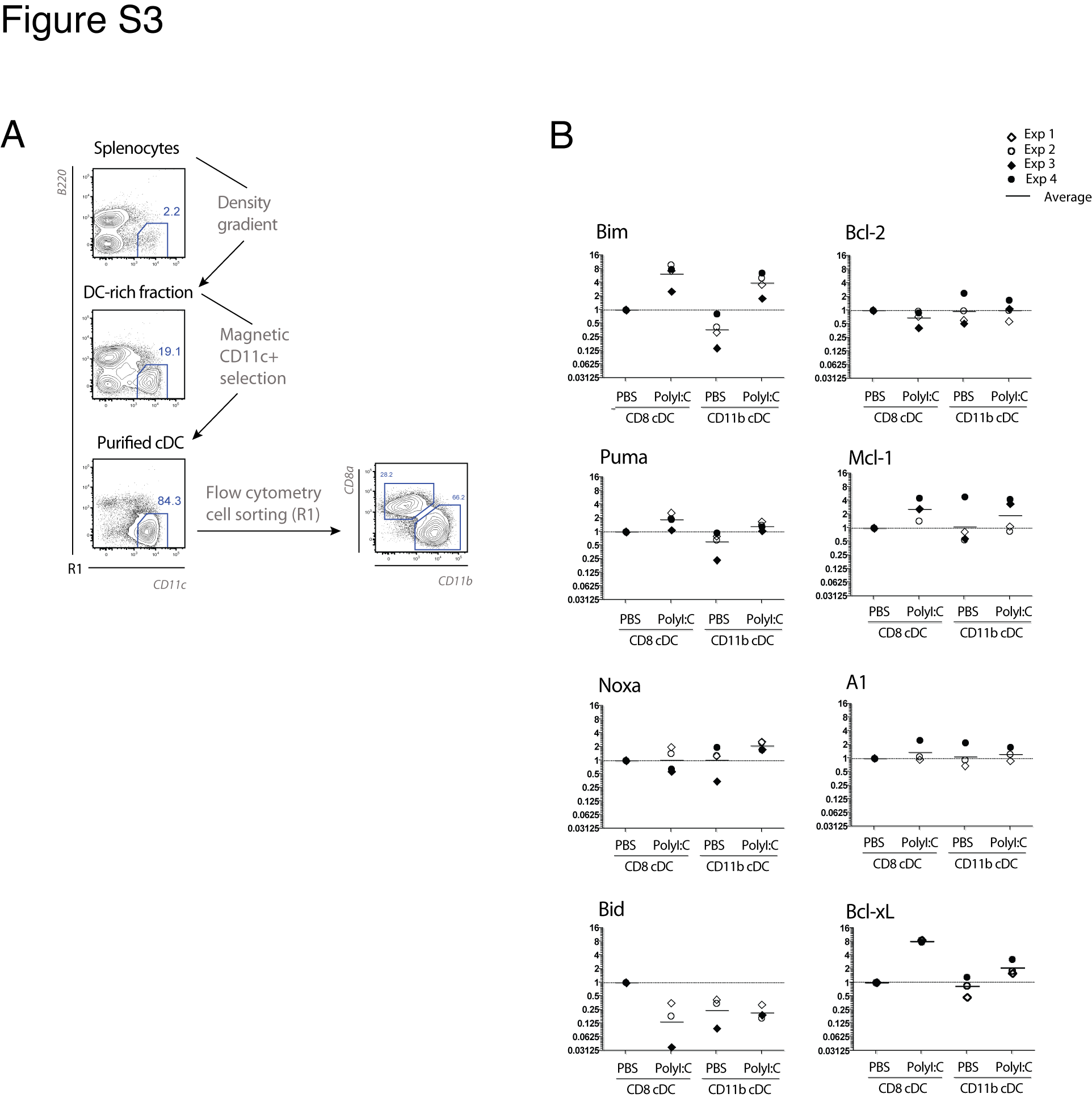

Supplement: Figure S3 — Changes in gene expression of Bcl-2 family members in cDC treated with PolyIC in vivo . Mice were injected with 50 ug PolyIC (n = 9) or PBS (control; n = 9). After 14 h, the spleens were harvested and pooled for cDC subset isolation and gene expression analysis. The experiment was performed twice in two independent laboratories as described in the Methods section, with a total of four biological replicates per sample. a. Strategy for flow cytometric analysis, gating and isolation of CD8a or CD11b cDC subsets from total splenocytes. One representative experiment is shown. b. Analysis of the levels of mRNA for pro-apoptotic Bcl-2 family members Bim (n = 4), Puma (n = 4), Noxa (n = 4), Bid (n = 3) and anti-apoptotic Bcl-2 (n = 4), Mcl-1 (n = 4), A1 (n = 3) and Bcl-xL (n = 3). Open symbols (○, ◊) correspond to qRT-PCR data generated at the WEHI (Melbourne), normalized to β-actin as a house-keeping gene. Closed symbols (•, ♦) correspond to qRT-PCR data generated at the DB-UNIL (Lausanne), normalized to TBP as a house-keeping gene. The primers used were as detailed in Table S2A. In order to obtain an inter-experimental and inter-laboratory comparison, a second normalization was performed relative to CD8a cDCs treated with PBS. (TIF) [file pone.0020189.s003.tif]

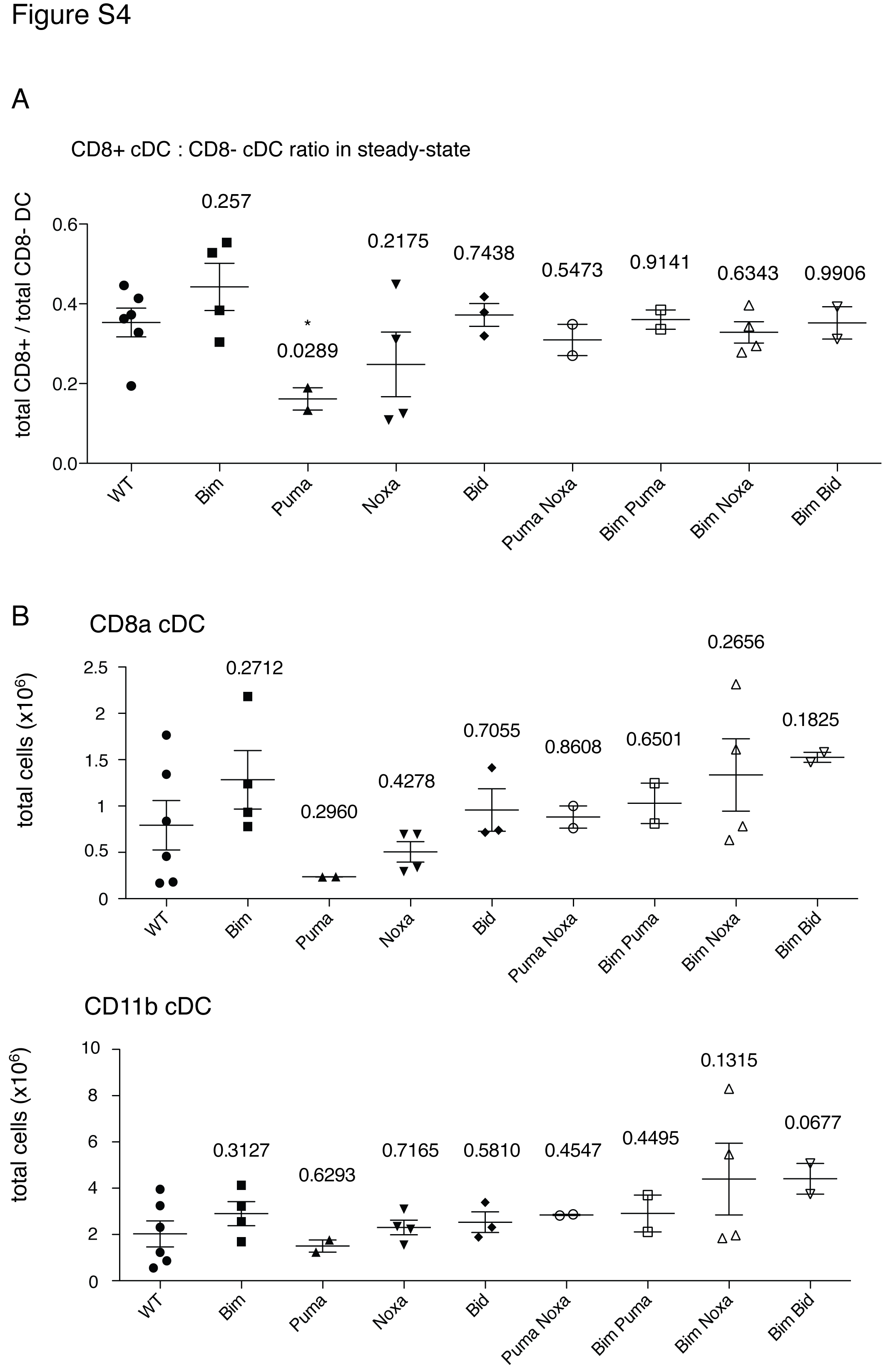

Supplement: Figure S4 — Steady-state splenic cDC populations in mice deficient for BH3-only members. Splenic cDC subsets were quantitated as described before amongst WT (n = 6), Bim-/- (n = 4), Puma-/- (n = 2), Noxa-/- (n = 4), Bid-/- (n = 3), Puma-/-Noxa-/- (n = 2), Bim-/-Puma-/- (n = 2), Bim-/-Noxa-/- (n = 4), Bim-/-Bid-/- (n = 2). A. Ratio of CD8a+-to-CD8a− cDC per mouse strain as indicated. B. Total numbers of each cDC subset per spleen per mouse strain as indicated. Data are represented as mean +/- SD. P-values indicate significance per strain as compared to WT controls. (TIF) [file pone.0020189.s004.tif]

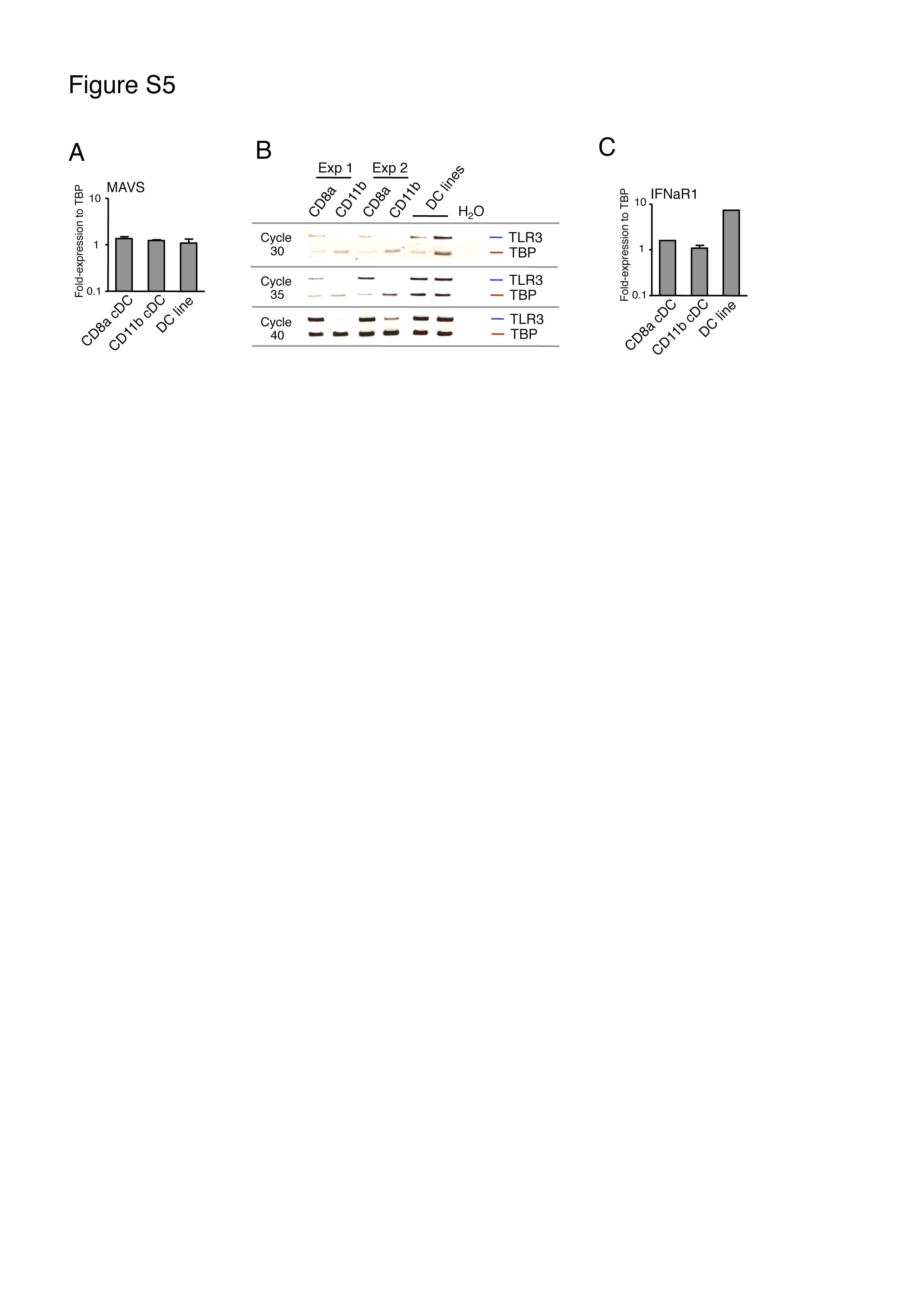

Supplement: Figure S5 — Expression levels for TLR 3, MAVS and the IFNaR1 in splenic cDC subsets and DC lines. Purified splenic CD8α as well as CD11b cDC subsets and DC line samples (n = 2 per sample) were examined for the levels of mRNA for Mavs and Ifnar1 by qRT-PCR using the primers detailed in Table S2B. Tlr3 mRNA expression was analyzed by semi-quantitative PCR. (TIF) [file pone.0020189.s005.tif]

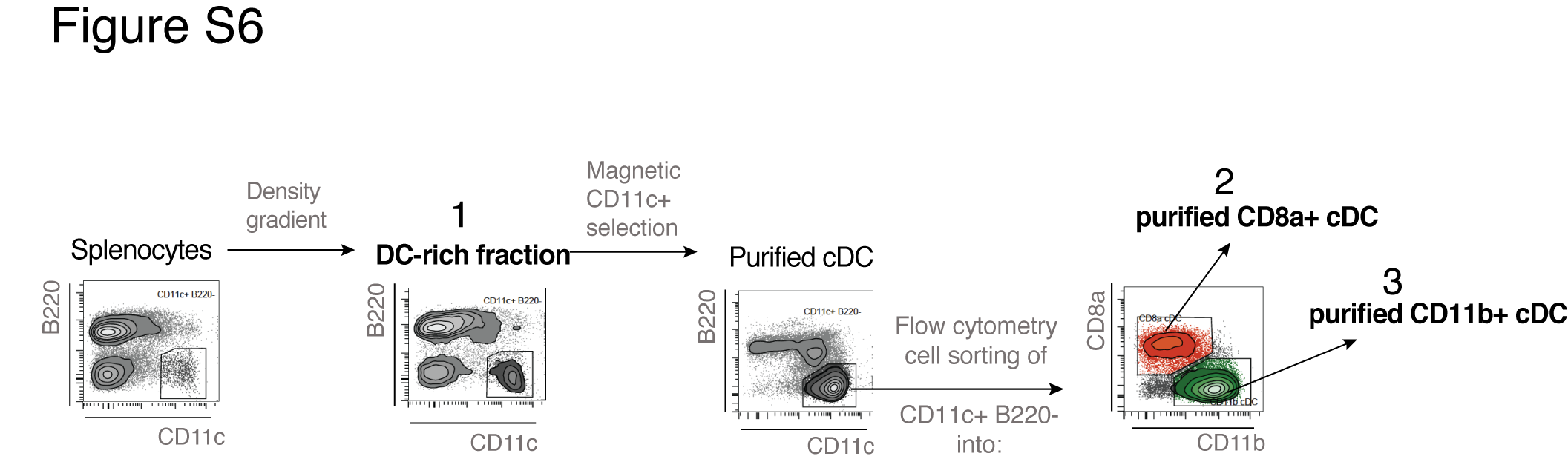

Supplement: Figure S6 — Strategy and flow cytometry analysis for the purification of DC-rich and cDC subset fractions. Splenocyte suspensions were fractionated by density centrifugation in isohexol carbohydrate medium (Nycodenz, Axis-Shield, Norway) at 1.077g/cm3. The light density fraction was collected as the DC-rich fraction (fraction 1, as indicated). CD11c+ cDC were isolated from DC-rich fractions using anti-CD11c antibody coupled magnetic micro-beads (Miltenyi Biotech). Purified cDC subsets were obtained from cDC by flow cytometry cell sorting separating CD11c+/B220− cells into CD8a or CD11b subsets, collecting fractions 2 and 3, respetively, as indicated. One representative sample is shown. The cDC represent 1-2% of total splenic leukocytes. Within the cDC population, typically ∼20% were CD8a+ cDC and ∼60% CD11b cDC. At least 5 mice were required to isolate ∼0.5×10^6^ CD8a+ cDC. Density centrifugation allowed for a ∼10-fold enrichment in DC prior to immunomagnetic bead selection. Enriched DC preparations subjected to flow-cytometric cell sorting contained >75% cDC, and resulting cDC subsets were purified to at least 95%. (TIF) [file pone.0020189.s006.tif]

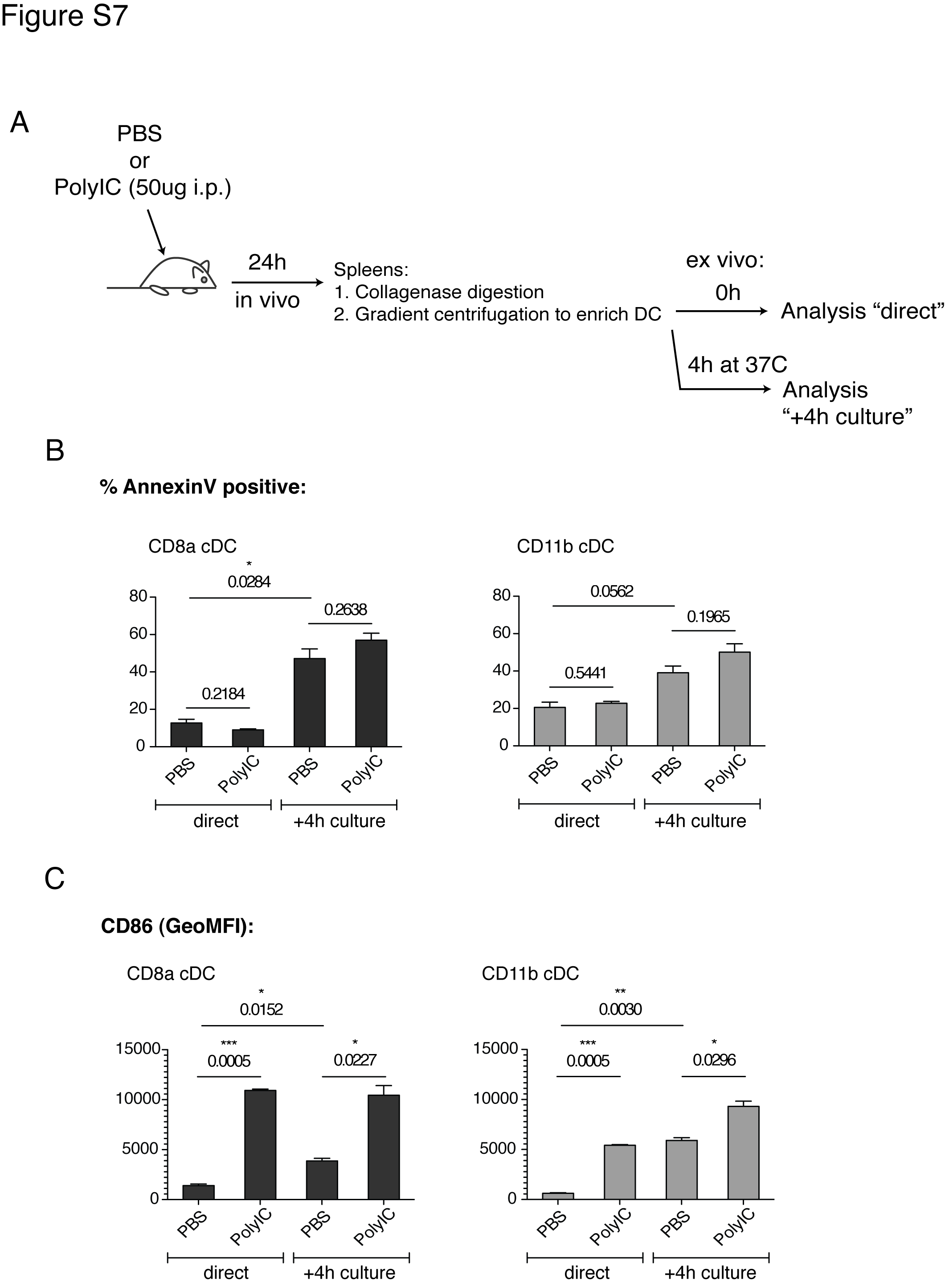

Supplement: Figure S7 — Activation and apoptosis in splenic cDC ex-vivo, isolated from either PBS- or PolyIC-treated mice. A. Experimental outline. Briefly, WT mice (n = 2) were treated with PBS or PolyIC for 24h. Splenic cDC were enriched by density centrifugation and analyzed inmediately (“0h”) or after 4h of culture (“4h”). Analysis included the segregation of cDC subsets as before and the apoptosis staining AnnexinV (B) or the activation marker CD86 (C). Data are represented as mean +/- SEM. (TIF) [file pone.0020189.s007.tif]

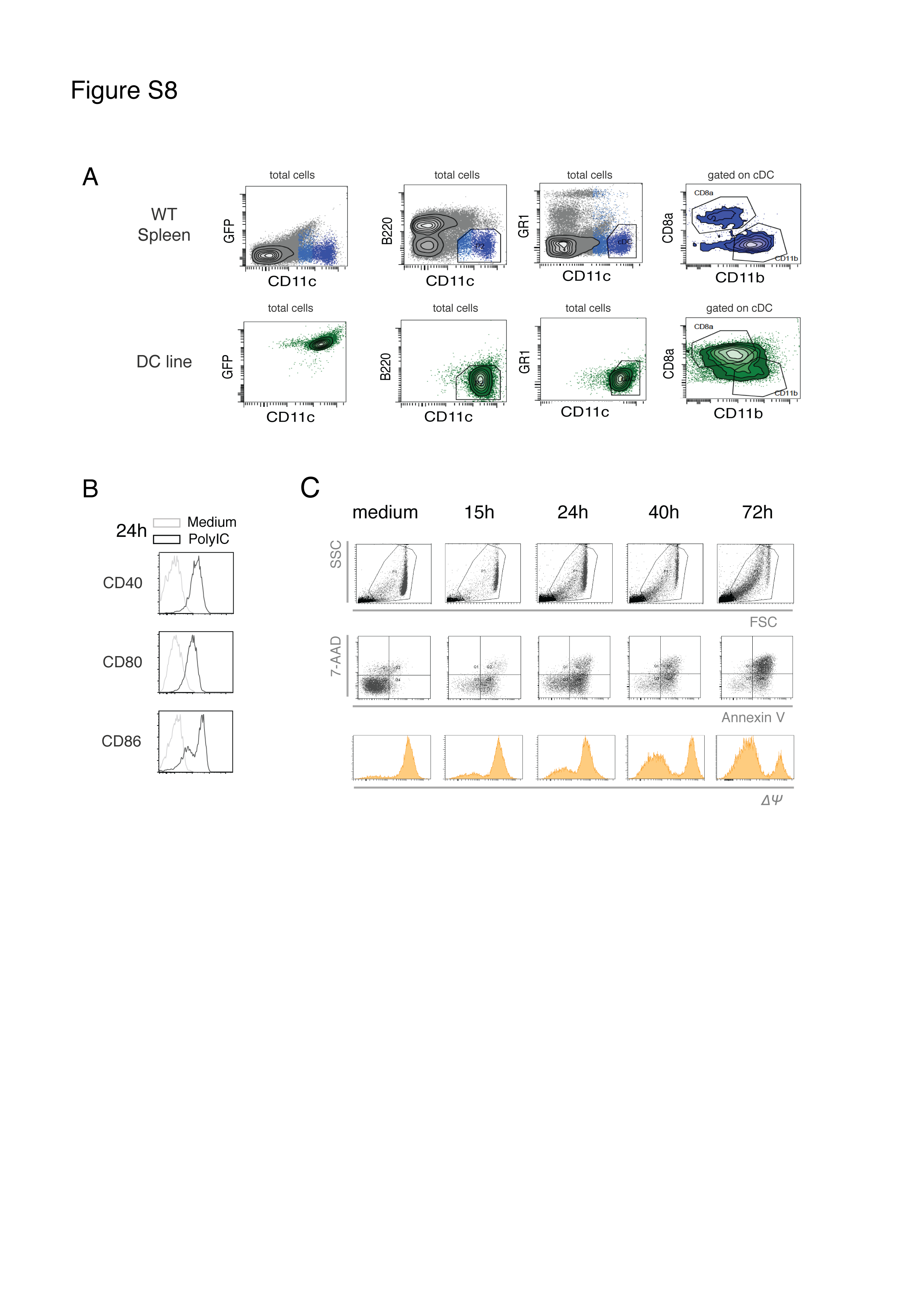

Supplement: Figure S8 — PolyIC induces activation and apoptosis in DC lines. A. Phenotype of DC lines derived from CD11c:SV40LgT-transgenic mice (NB. harbouring the eGFP reporter for the transgene) [23], analyzed by flow cytometry and compared to splenic cDC in WT mice as indicated. B and C. DC lines (WT) were treated in vitro with 8 ug/mL poly I:C. B. Histograms showing the up-regulation of co-stimulatory markers CD40, CD80 and CD86 at 24 h of treatment with PolyIC (dark grey) as compared to unteated controls (pale grey). C. The kinetics of poly I:C treatment are shown for a representative experiment, with flow cytometry analysis of FSC vs SSC plots, AnnexinV/7-AAD double-staining and TMRM histograms at different time points as indicated. (TIF) [file pone.0020189.s008.tif]

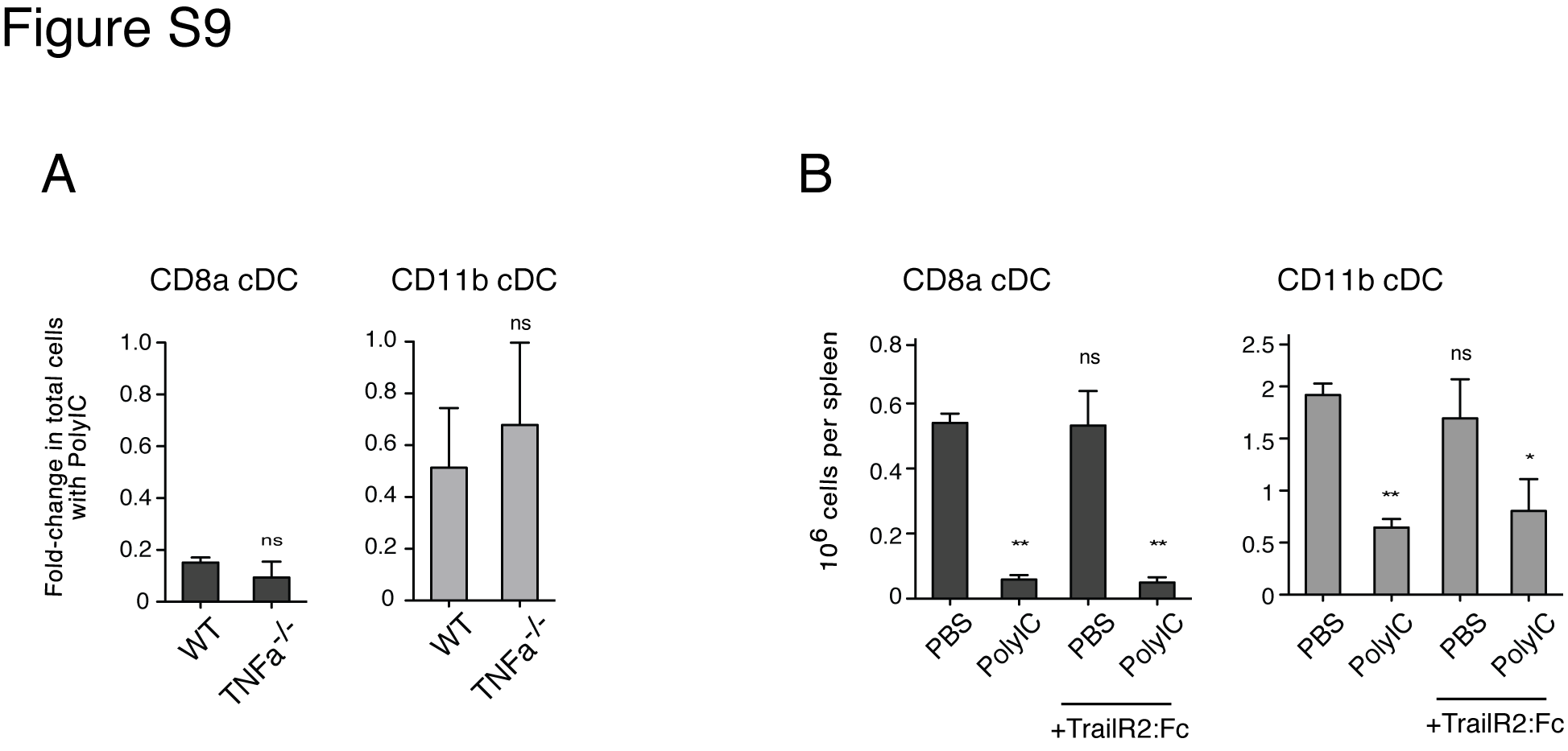

Supplement: Figure S9 — Splenic cDC loss with PolyIC still occurs in TNFa-/- mice or with TRAIL-R2:Fc blockade. A. WT and TNFa -/- mice were treated with PBS (control) or PolyIC and splenic cDC were analyzed at 40 h as described before (n = 2 per treatment per mouse strain). The fold-change in total cDC subset cells with PolyIC relative to PBS treatment is shown per strain as indicated. B. WT mice were treated with PBS or TRAIL-R2:Fc 6h before and at 18h after treatment with either PBS or PolyIC (time 0h) and splenic cDC were analyzed at 40 h as described before (n = 2 per treatment). Total numbers of each cDC subset per are shown per treatment as indicated. Data are presented as mean +/- SD. (TIF) [file pone.0020189.s009.tif]
