# Supplementary material for: Type I Interferon Drives Dendritic Cell Apoptosis via Multiple BH3-Only Proteins following Activation by PolyIC In Vivo
Source: PLoS One. 2011 Jun 2;6(6):e20189. doi: 10.1371/journal.pone.0020189 (PMC3107228; doi:10.1371/journal.pone.0020189)
Supplement: Materials and Methods S1 — Materials and Methods used for the experiments shown in Supporting Figures and related to Materials and Methods. (DOC) [file pone.0020189.s010.doc]

**SUPPORTING INFORMATION: MATERIALS AND METHODS S1. Materials and Methods used for the experiments shown in Supporting Figures and related to Materials and Methods.**

**Mice and treatments.** Additional mice used for experiments in supporting figures were females or males aged at least 8 weeks. Mice were WT (C57BL/6) or *Rag2^-/-^γc^-/-^,* or *Tnfa^-/-^* (see strain nomenclature, genetic background and references in Table S1). Mice were injected intra-peritoneally (i.p.) with 50 ug PolyIC (TLR3-L from Invivogen) or, as a control, with PBS. Other TLR-Ls injected were 50 ug phospho-thioester-CpG (ODN1826 from TriLink BioTech) and 10 ug LPS (Ultra Pure E. coli LPS from InvivoGen). TRAIL blockade in vivo consisted in treatment with human TRAIL-R2:Fc (cross-reacting with mouse TRAIL[1]), performing two injections i.v., each with 200ug of TRAIL-R2:Fc, one 6h before and the second 18h after treatment with PBS or PolyIC (time 0h). TRAIL-R2:Fc was produced as previously described [2].

**cDC subset purification for qRT-PCR analysis.** The experiment was performed twice in two independent laboratories, DB-UNIL (Lausanne, CH) and WEHI (Melbourne, AUS). Following collagenase D and DNAse I digestion of spleens, as described above, total splenocytes were fractionated by density centrifugation in isohexol carbohydrate medium (Nycodenz, Axis-Shield, Norway) at 1.077g/cm3 [3]. The DC-enriched, light density fraction was collected and washed in PBS containing 5 mM EDTA prior to enrichment of CD11c DCs using anti-CD11c antibody coupled magnetic micro-beads (Miltenyi Biotech) for the experiments at DB-UNIL (Lausanne, CH) or negative selection of CD11c DCs using a biotinylated antibody cocktail and streptavidin-conjugated magnetic microbeads (Dynal, Invitrogen) at the WEHI (Melbourne, AUS). Enriched DCs were stained with surface marker-specific antibodies for flow cytometric cell sorting of CD8a or SIRPa/CD11b DC subsets[3,4]. CD11c/CD45RA^-^ cells were sorted for CD8a or SIRPa subsets using Mo-Flo cell sorter (Beckman Coulter) at the WEHI (Melbourne, AUS). CD11c/B220^-^ cells were sorted to CD8a or CD11b subsets with a FACSAria cell sorter (Becton Dickinson) at the LICR (Lausanne, CH). The cDCs represent 1-2% of total splenic leukocytes. Within the cDC population, typically ~20% were CD8a cDCs and ~60% CD11b cDCs. At least 9 mice were required to isolate 1.5x10^6^ CD8a cDCs. Density centrifugation allowed for a ~10-fold enrichment in DCs prior to immunomagnetic bead selection. Enriched DC preparations subjected to flow-cytometric cell sorting contained >85% cDCs, and resulting cDC subsets were purified to at least 95%.

**Quantitative Real-time PCR (qRT-PCR) analysis of Bcl-2 family genes.** Purifed cDC subsets were immediately lysed for total RNA isolation using either spin columns from Qiagen RNeasy® following the manufacturer’s protocol (DB-UNIL, Lausanne, CH) or Trizol extraction (WEHI, Lausanne, CH). DNAse digestion was performed on the columns. Total RNA yields were quantified by Nanodrop spectrophotometry (Thermo Fisher Scientific Inc.). Synthesis of cDNA was performed using random nonamer primers and Superscript II Reverse Transcriptase (Invitrogen) as previously described[5] (DB-UNIL, Lausanne, CH) or using the TaqMan RT system (Roche) (WEHI, Melbourne, AUS). Quantitative real-time PCR was performed using SYBR Green mix on LighCycler (Roche Diagnostics) (DB-UNIL, Lausanne, CH) or the SYBR Green PCR Kit (Qiagen) on ABI Prism 7900 (Applied Biosystems) (WEHI, Melbourne, AUS). The genes analyzed were *Bim*, *Puma*, *Noxa*, *Bid*, *Bcl-2*, *Mcl-1*, *A1* and *Bcl-xL*. The primers used were as listed in Table S2A. Relative expression levels were analyzed using second derivative method with LC data analysis 3.5Software (Roche). House-keeping genes were TBP (DB-UNIL, Lausanne, CH) or b-actin (WEHI, Melbourne, AUS). QRT-PCR was performed with at least technical duplicates. For the analysis, expression of each gene was normalized to the house-keeping gene, generating a single value per biological replicate. In order to obtain an inter-experimental and inter-laboratory comparison, a second normalization was performed relative to CD8a cDCs treated with PBS.

**Gene expression analysis of MAVS, TLR3 and IFNaR1.**

***RNA isolation and cDNA synthesis.*** Total RNA was isolated from DC line samples or purified splenic cDC subsets using the spin columns from Qiagen RNeasy® following the manufacturer’s protocol, with DNAse I digestion on-column. Synthesis of cDNA was performed using random nonamer primers and the Superscript II Reverse Transcriptase kit (Invitrogen). Cleanup of cDNA was performed using spin columns from Wizard SV Gel and PCR cleanup (Promega). CDNA Samples were diluted at 3 ng/uL for q-RTPCR or at 50 ng/uL for semi-quantitative PCR, using 2 uL per PCR reaction (5 uL and 10 uL final volume, respectively).

***Quantitative Real-time PCR (q-RTPCR) and semi-quantitative PCR analysis****.* The genes analyzed and corresponding primers were as listed in Table S2B. Q-RTPCR was performed for *Mavs* and *Ifnar1* using SYBR Green mix on LighCycler480 (384-well plate, 5 uL reaction) from Roche Diagnostics. Primers for *Mavs* were designed against the reference sequence NM_144881.1. The specificity of the reaction was verified by loading the q-RTPCR product on gel to check for generation of the unique size-specific band and absence of primer-dimers, and by restriction enzyme digestion of the q-RTPCR product. Analysis of q-RTPCR for Ifnar1 was performed using the primers for the genotyping of *Ifnar1*^-/-^ mice (WT control PCR), with the q-RTPCR product verified on gel. CDNA Titrations were performed in order to control for the linearity between the target gene and the house-keeping gene. Relative expression levels were analyzed using second derivative method with LC data analysis 3.5Software (Roche). The house-keeping gene used was TBP. Q-RTPCR was performed with at least technical duplicates. For the analysis, expression of each gene was normalized to the house-keeping gene, generating a single value per biological replicate.

*Tlr3* expression was analyzed by semi-quantitative PCR under standard PCR conditions and stopping the reaction after 30, 35 and 40 cycles and visualizing amplicons on gel. TBP and TLR3 reactions were run on the same sample for simultaneous visualization on gel.

**Derivation of Splenic DC lines*.***

DC lines were derived from previously described CD11c:SV40LgT- transgenic mice [6]. Spontaneous immortalization of tumoral CD8a DC was allowed *in vitro* by culture in complete medium and in the absence of growth factors. Total splenocytes from tumor-burdened transgenic animals were seeded in serial dilutions beginning at high densities (>20x10^6^ cells / mL). Selection of adherent DC and removal of dead cells were performed by periodic change of medium. The splitting dilution at early passages was at a maximum 1:2, and increased as DC tolerated cell splitting better. DC lines were generally stable and readily used by passage 10. The derivation process lasted from two months to over half a year, with most DC lines derived within 4 months. DC lines lacking genes of interest (*Tlr3*, *Mavs* and *Ifnar1*) were generated by crossing the CD11c:SV40-LgT-Tg mice[6] to the relevant gene-targeted mice. DC line cells were harvested by incubation in non-enzymatic, 5mM EDTA-based buffer and split 1:10 to 1:15 from confluency. Of note, DC lines are GFP positive. The generation and use of mouse DC lines represents a considerable advance in the efforts towards the implementation of the 3R (Refine, Reduce, Replace) principle in animal experimentation.

**Mitochondrial depolarization (TMRM staining).** Staining of live mitochondria was performed by incubating DC lines in 100 nM tetramethyrhodamine methyl ester (TMRM) for 20 min at 37C. Cells were then harvested for immediate flow cytometry analysis of TMRM signal in the PE channel.

**Lentiviral transduction of DC lines.**

Second generation lentiviral plasmids used were (pWP-SIN-cPPT-WPRE)-CMV-IRES-GFP lentiviral vector and the two packaging plasmids pMD2G and psPAX2. The lentiviral system and protocols have been described elsewhere[7]. The IFNβ insert (NM_010510) was obtained by PCR amplification of cDNA from WT DC lines stimulated with poly IC. Transduction was done into 1IFNR-/- DC lines, performing either mock (CMV:GFP; control) or IFNβ (CMV:IFNβ:GFP) transductions. The MOI used was between 5-10. Efficiency of transduction was measured by IFNβ ELISA on cell culture supernatants, with yields in the range of 10-100ng/mL.

**Materials and Methods S1: References**

1. Bossen C, Ingold K, Tardivel A, Bodmer JL, Gaide O, et al. (2006) Interactions of tumor necrosis factor (TNF) and TNF receptor family members in the mouse and human. J Biol Chem 281: 13964-13971.

2. Schneider P (2000) Production of recombinant TRAIL and TRAIL receptor: Fc chimeric proteins. Methods Enzymol 322: 325-345.

3. Naik SH, Metcalf D, van Nieuwenhuijze A, Wicks I, Wu L, et al. (2006) Intrasplenic steady-state dendritic cell precursors that are distinct from monocytes. Nat Immunol 7: 663-671.

4. Villadangos JA, Schnorrer P (2007) Intrinsic and cooperative antigen-presenting functions of dendritic-cell subsets in vivo. Nat Rev Immunol 7: 543-555.

5. Otten LA, Leibundgut-Landmann S, Huarte J, Kos-Braun IC, Lavanchy C, et al. (2006) Revisiting the specificity of the MHC class II transactivator CIITA in vivo. Eur J Immunol 36: 1548-1558.

6. Steiner QG, Otten LA, Hicks MJ, Kaya G, Grosjean F, et al. (2008) In vivo transformation of mouse conventional CD8alpha+ dendritic cells leads to progressive multisystem histiocytosis. Blood 111: 2073-2082.

7. Salmon P, Trono D (2007) Production and titration of lentiviral vectors. Curr Protoc Hum Genet Chapter 12: Unit 12 10.
