# Supplementary material for: Type I Interferon Drives Dendritic Cell Apoptosis via Multiple BH3-Only Proteins following Activation by PolyIC In Vivo
Source: PLoS One. 2011 Jun 2;6(6):e20189. doi: 10.1371/journal.pone.0020189 (PMC3107228; doi:10.1371/journal.pone.0020189)
Supplement: Table S1 — Mouse strains used (related to Materials and Methods and Materials and Methods S1). (DOC) [file pone.0020189.s011.doc]

**Table S1. Mouse strains used (related to Materials and Methods).**

| **Name** | **Official nomenclature** | **Reference** |
| --- | --- | --- |
| Bim^-/-^ | C57BL/6-*Bcl2l11^tm1.1Ast^*/J (*) | [1] |
| Puma^-/-^ | C57BL/6-*Bbc3^tm1.Ast^*(*) | [2] |
| Noxa^-/-^ | C57BL/6-*Pmaip1^tm1.Ast^*(*) | [2] |
| Bid^-/-^ | C57BL/6-*Bid^tm1.Sjk^*(*) | [3] |
| Puma^-/-^Noxa^-/-^ | C57BL/6-*Bbc3^tm1.Ast^* x  C57BL/6-*Pmaip1^tm1.Ast^* |  |
| Bim^-/-^Bid^-/-^ | C57BL/6-*Bcl2l11^tm1.1Ast^*/J x  C57BL/6-*Bid^tm1.Sjk^* | [4] |
| Bim^-/-^Puma^-/-^ | C57BL/6-*Bcl2l11^tm1.1Ast^*/J x  C57BL/6-*Bbc3^tm1.Ast^* | [5] |
| Bim^-/-^Noxa^-/-^ | C57BL/6-*Bcl2l11^tm1.1Ast^*/J x C57BL/6-*Pmaip1^tm1.Ast^* | [6] |
| Perforin^-/-^ | C57BL/6-Prf1^tm1Sdz^/J | [7] |
| FAS mutant | MRL/MpJ-Fas^lpr^ | [8] |
| Rag2^-/-^γc^-/-^ | C57BL6/JxC57BL/10SgSnAi | [9] |
| TLR3-/- | B6;129S1-tlr3tm1Flv/J | [10] |
| MAVS-/- | C57Bl/6-TgH(CARDIF)604Siec | [11] |
| TLR3-/- MAVS-/- | B6;129S1-tlr3tm1Flv/Jx C57Bl/6-TgH(CARDIF)604Siec |  |
| IFNaR-/- | C57BL/6.Ifnar1tm1/Agt | [12] |
| TNFa-/- | BL6.129S-Tnftm1/Ljo | [13] |

(*) back-crossed to C57BL/6 for at least 20 generations.

**Table S1: references**

1. Bouillet P, Metcalf D, Huang DC, Tarlinton DM, Kay TW, et al. (1999) Proapoptotic Bcl-2 relative Bim required for certain apoptotic responses, leukocyte homeostasis, and to preclude autoimmunity. Science 286: 1735-1738.

2. Villunger A, Michalak EM, Coultas L, Mullauer F, Bock G, et al. (2003) p53- and drug-induced apoptotic responses mediated by BH3-only proteins puma and noxa. Science 302: 1036-1038.

3. Kaufmann T, Tai L, Ekert PG, Huang DC, Norris F, et al. (2007) The BH3-only protein bid is dispensable for DNA damage- and replicative stress-induced apoptosis or cell-cycle arrest. Cell 129: 423-433.

4. Willis SN, Fletcher JI, Kaufmann T, van Delft MF, Chen L, et al. (2007) Apoptosis initiated when BH3 ligands engage multiple Bcl-2 homologs, not Bax or Bak. Science 315: 856-859.

5. Erlacher M, Labi V, Manzl C, Bock G, Tzankov A, et al. (2006) Puma cooperates with Bim, the rate-limiting BH3-only protein in cell death during lymphocyte development, in apoptosis induction. J Exp Med 203: 2939-2951.

6. Huntington ND, Puthalakath H, Gunn P, Naik E, Michalak EM, et al. (2007) Interleukin 15-mediated survival of natural killer cells is determined by interactions among Bim, Noxa and Mcl-1. Nat Immunol 8: 856-863.

7. Kagi D, Ledermann B, Burki K, Seiler P, Odermatt B, et al. (1994) Cytotoxicity mediated by T cells and natural killer cells is greatly impaired in perforin-deficient mice. Nature 369: 31-37.

8. Nagata S, Suda T (1995) Fas and Fas ligand: lpr and gld mutations. Immunol Today 16: 39-43.

9. Mazurier F, Fontanellas A, Salesse S, Taine L, Landriau S, et al. (1999) A novel immunodeficient mouse model--RAG2 x common cytokine receptor gamma chain double mutants--requiring exogenous cytokine administration for human hematopoietic stem cell engraftment. J Interferon Cytokine Res 19: 533-541.

10. Alexopoulou L, Holt AC, Medzhitov R, Flavell RA (2001) Recognition of double-stranded RNA and activation of NF-kappaB by Toll-like receptor 3. Nature 413: 732-738.

11. Sun Q, Sun L, Liu HH, Chen X, Seth RB, et al. (2006) The specific and essential role of MAVS in antiviral innate immune responses. Immunity 24: 633-642.

12. Muller U, Steinhoff U, Reis LF, Hemmi S, Pavlovic J, et al. (1994) Functional role of type I and type II interferons in antiviral defense. Science 264: 1918-1921.

13. Marino MW, Dunn A, Grail D, Inglese M, Noguchi Y, et al. (1997) Characterization of tumor necrosis factor-deficient mice. Proc Natl Acad Sci U S A 94: 8093-8098.
