# Supplementary material for: Type I Interferon Drives Dendritic Cell Apoptosis via Multiple BH3-Only Proteins following Activation by PolyIC In Vivo
Source: PLoS One. 2011 Jun 2;6(6):e20189. doi: 10.1371/journal.pone.0020189 (PMC3107228; doi:10.1371/journal.pone.0020189)
Supplement: Table S2 — Primers used for gene expression analyses (related to Materials and Methods S1). (DOC) [file pone.0020189.s012.doc]

**Table S2. Primers used for gene expression analyses (related to Materials and Methods).**

**A. Primers used for gene expression analyses of Bcl-2 family members and house-keeping genes.**

Primers used at DB-UNIL, Lausanne, CH.

| **Gene** | **Primer** | **Sequence** |  | **Product** |
| --- | --- | --- | --- | --- |
|  |  |  |  |  |
| **Bim** | sense | GAGTTGTGACAAGTCAACACAAACC | Tm = 62.1 | 256 |
|  | antisense | GAAGATAAAGCGTAACAGTTGTAAGATAACC | Tm = 62.0 |  |
| **Puma** | sense | ATGGCGGACGACCTCAAC | Tm = 64.1 | 106 |
|  | antisense | GGAGTCCCATGAAGAGAT | TM = 60.1 |  |
| **Noxa** | sense | ACTGTGGTTCTGGCGCAGAT | Tm = 63.18 | 150 |
|  | antisense | TTGAGCACACTCGTCCTTCAA | Tm = 61.95 |  |
| **Bid** | sense | TCTGAAAGTCAGGAAGAAATCATCC | Tm = 62.1 | 313 |
|  | antisense | GGTTAATAAAGTTGACAGTCGTGTGG | Tm = 62.1 |  |
| **Bcl-2** | sense | GCTACCGTCGTGACTT | Tm = 59.5 | 325 |
|  | antisense | AGCCAGGAGAAATCAAACA | Tm = 59.9 |  |
| **Mcl-1** | sense | AGAGCGCTGGAGACCCTG | Tm = 61.71 | 430 |
|  | antisense | CTATCTTATTAGATATGCCAGACC | Tm = 53.47 |  |
| **A1** | sense | GTCATACTTGGATGACTTTCACGTG | Tm = 62.0 | 239 |
|  | antisense | ATTCTCCTGTGTTATTCATTATGAATTCTG | Tm = 62.0 |  |
| **Bcl-xL** | sense | TGGAGTCAGTTTAGTGATGTCGAAG | Tm = 61.9 | 340 |
|  | antisense | AGTTTACTCCATCCCGAAAGAGTTC | Tm = 62.2 |  |
| **TBP** | sense | CCTTCACCAATGACTCCTATGAC | Tm = 59.88 | 118 |
|  | antisense | CAAGTTTACAGCCAAGATTCAC | Tm = 56.61 |  |

Primers used at WEHI, Melbourne, AUS.

| **Gene** | **Primer** | **Sequence** |  | **Product** |
| --- | --- | --- | --- | --- |
|  |  |  |  |  |
| **Bim** | sense | GAGTTGTGACAAGTCAACACAAACC | Tm = 62.1 | 256 |
|  | antisense | GAAGATAAAGCGTAACAGTTGTAAGATAACC | Tm = 62.0 |  |
| **Puma** | sense | ATGCCTGCCTCACCTTCATCT | Tm = 63.36 | 61 |
|  | antisense | AGCACAGGATTCACAGTCTGGA | Tm = 62.20 |  |
| **Noxa** | sense | ACTGTGGTTCTGGCGCAGAT | Tm = 63.18 | 102 |
|  | antisense | TTGAGCACACTCGTCCTTCAA | Tm = 61.95 |  |
| **Bid** | sense | TCTGAAAGTCAGGAAGAAATCATCC | Tm = 62.1 | 313 |
|  | antisense | GGTTAATAAAGTTGACAGTCGTGTGG | Tm = 62.1 |  |
| **Bcl-2** | sense | TTATAAGCTGTCACAGAGGGGCTAC | Tm = 62.07 | 391 |
|  | antisense | GAACTCAAAGAAGGCCACAATCCTC | Tm = 65.47 |  |
| **Mcl-1** | sense | AGAGCGCTGGAGACCCTG | Tm = 61.71 | 430 |
|  | antisense | CTATCTTATTAGATATGCCAGACC | Tm = 53.47 |  |
| **A1** | sense | GTCATACTTGGATGACTTTCACGTG | Tm = 62.0 | 239 |
|  | antisense | ATTCTCCTGTGTTATTCATTATGAATTCTG | Tm = 62.0 |  |
| **Bcl-xL** | sense | TGGAGTCAGTTTAGTGATGTCGAAG | Tm = 61.9 | 340 |
|  | antisense | AGTTTACTCCATCCCGAAAGAGTTC | Tm = 62.2 |  |
| **b-Actin** | sense | TATTGGCAACGAGCGGTTC | Tm = 62.10 | 62 |
|  | antisense | CCATACCCAAGAAGGAAGGCT | Tm = 61.67 |  |

**B. Primers used for gene expression analyses of tlr3, mavs, ifnar1 and house-keeping genes.**

| ***Semi-quantitative PCR for TLR3 (3mM Mg, 60C annealing, 15'' at 72C) (TLR3 and TBP run together)*** | | | | | |  |
| --- | --- | --- | --- | --- | --- | --- |
| **Target** | **Primer** | **Sequence** | **Tm** | | **Product** | **Reference** |
| **TLR3** | Forward | GAGGGCTGGAGGATCTCTTT | 52.55 | | 351 | [1] |
|  | Reverse | TGCCTCAATAGCTTGCTGAA | 51.66 | |  |  |
| **TBP** | Forward | CCACAGGCTCACCCATACTTC | 61.84 | | 129 | in house |
|  | Reverse | GGGATGTCCTAGGTGGTGACA | 62.14 | |  |  |
|  |  |  |  | |  |  |
| ***Primers on LightCycler480 (384 plate, 5ul), STD Prog: 60C annealing, 10'' at 72C, no additional Mg*** | | | | | |  |
| **Target** | **Primer** | **sequence** | **Tm** | **Product** | | **Reference** |
| **MAVS** | Forward | GCTGCCACCTGTTTCAGTAC | 58.37 | 159 | | in house |
|  | Reverse | TGGCGCTGTATTGGTGAGC | 63.33 |  | |  |
| **IFNaR** | Forward | AAG ATG TGC TGT TCC CTT CCT CTG CTC TGA | 73.01 | 151 | | [2] |
|  | Reverse | ATT ATT AAA AGA AAA GAC GAG GCG AAG TGG | 66.89 |  | |  |
| **TBP** | Forward | CCTTCACCAATGACTCCTATGAC | 59.88 | 118 | | in house |
|  | Reverse | CAAGTTTACAGCCAAGATTCAC | 56.51 |  | |  |

**Table S2: References**

1. Edwards AD, Diebold SS, Slack EM, Tomizawa H, Hemmi H, et al. (2003) Toll-like receptor expression in murine DC subsets: lack of TLR7 expression by CD8 alpha+ DC correlates with unresponsiveness to imidazoquinolines. Eur J Immunol 33: 827-833.

2. Muller U, Steinhoff U, Reis LF, Hemmi S, Pavlovic J, et al. (1994) Functional role of type I and type II interferons in antiviral defense. Science 264: 1918-1921.
